# PeptiCHIP: A Microfluidic Platform for Tumor Antigen Landscape Identification

**DOI:** 10.1021/acsnano.1c04371

**Published:** 2021-10-04

**Authors:** Sara Feola, Markus Haapala, Karita Peltonen, Cristian Capasso, Beatriz Martins, Gabriella Antignani, Antonio Federico, Vilja Pietiäinen, Jacopo Chiaro, Michaela Feodoroff, Salvatore Russo, Antti Rannikko, Manlio Fusciello, Satu Koskela, Jukka Partanen, Firas Hamdan, Sari M. Tähkä, Erkko Ylösmäki, Dario Greco, Mikaela Grönholm, Tuija Kekarainen, Masoumeh Eshaghi, Olga L. Gurvich, Seppo Ylä-Herttuala, Rui M. M. Branca, Janne Lehtiö, Tiina M. Sikanen, Vincenzo Cerullo

**Affiliations:** † Drug Research Program (DRP), ImmunoViroTherapy Lab (IVT), Division of Pharmaceutical Biosciences, Faculty of Pharmacy, University of Helsinki, Viikinkaari 5E, 00790 Helsinki, Finland; ‡ Helsinki Institute of Life Science (HiLIFE), University of Helsinki, Fabianinkatu 33, 00710 Helsinki, Finland; § Translational Immunology Program (TRIMM), Faculty of Medicine Helsinki University, University of Helsinki, Haartmaninkatu 8, 00290 Helsinki, Finland; ∥ Digital Precision Cancer Medicine Flagship (iCAN), University of Helsinki, 00014 Helsinki, Finland; ⊥ Drug Research Program, Division of Pharmaceutical Chemistry and Technology, Faculty of Pharmacy, University of Helsinki, Viikinkaari 5E, 00790 Helsinki, Finland; # Faculty of Medicine and Health Technology, Tampere University, Arvo Ylpön katu 34, Tampere 33520, Finland; × Institute for Molecular Medicine Finland, FIMM, Helsinki Institute of Life Science (HiLIFE), University of Helsinki, Biomedicum 2U, Tukholmankatu 8, 00290 Helsinki, Finland; + Department of Urology, Helsinki University and Helsinki University Hospital, Haartmaninkatu 8, 00029 Helsinki, Finland; ⊗ Research Program in Systems Oncology, Faculty of Medicine, University of Helsinki, Haartmaninkatu 8, 00029 Helsinki, Finland; $ Research & Development Finnish Red Cross Blood Service Helsinki, Kivihaantie 7, 00310 Helsinki, Finland; ¶ Kuopio Center for Gene and Cell Therapy, Microkatu 1S, 70210 Kuopio, Finland; ○ A. I. Virtanen Institute, University of Eastern Finland, Neulaniementie 2, 70211 Kuopio, Finland; △ Science for Life Laboratory, Department of Oncology-Pathology, Karolinska Institutet, Tomtebodavagen 23B, 171 21 Solna, Sweden; ● Department of Molecular Medicine and Medical Biotechnology, Naples University “Federico II”, S. Pansini 5, 80131 Naples, Italy

**Keywords:** ligandome, HLA peptides, affinity purification, microfluidics, thiol−enes

## Abstract

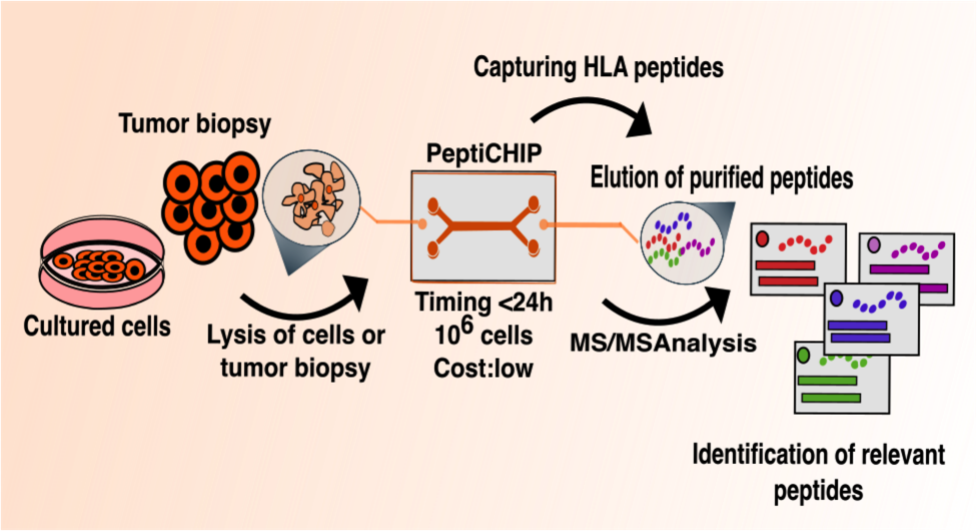

Identification of HLA class I ligands from the tumor surface (ligandome or immunopeptidome) is essential for designing T-cell mediated cancer therapeutic approaches. However, the sensitivity of the process for isolating MHC-I restricted tumor-specific peptides has been the major limiting factor for reliable tumor antigen characterization, making clear the need for technical improvement. Here, we describe our work from the fabrication and development of a microfluidic-based chip (PeptiCHIP) and its use to identify and characterize tumor-specific ligands on clinically relevant human samples. Specifically, we assessed the potential of immobilizing a pan-HLA antibody on solid surfaces *via* well-characterized streptavidin–biotin chemistry, overcoming the limitations of the cross-linking chemistry used to prepare the affinity matrix with the desired antibodies in the immunopeptidomics workflow. Furthermore, to address the restrictions related to the handling and the limited availability of tumor samples, we further developed the concept toward the implementation of a microfluidic through-flow system. Thus, the biotinylated pan-HLA antibody was immobilized on streptavidin-functionalized surfaces, and immune-affinity purification (IP) was carried out on customized microfluidic pillar arrays made of thiol–ene polymer. Compared to the standard methods reported in the field, our methodology reduces the amount of antibody and the time required for peptide isolation. In this work, we carefully examined the specificity and robustness of our customized technology for immunopeptidomics workflows. We tested this platform by immunopurifying HLA-I complexes from 1 × 10^6^ cells both in a widely studied B-cell line and in patients-derived *ex vivo* cell cultures, instead of 5 × 10^8^ cells as required in the current technology. After the final elution in mild acid, HLA-I-presented peptides were identified by tandem mass spectrometry and further investigated by *in vitro* methods. These results highlight the potential to exploit microfluidics-based strategies in immunopeptidomics platforms and in personalized immunopeptidome analysis from cells isolated from individual tumor biopsies to design tailored cancer therapeutic vaccines. Moreover, the possibility to integrate multiple identical units on a single chip further improves the throughput and multiplexing of these assays with a view to clinical needs.

## Introduction

Cancer immunotherapy relies on the priming of T cells, the generation and stimulation of cytotoxic CD8 T lymphocytes within the tumor microenvironment, and the establishment of an efficient and durable antitumor immune response.^1^ In this context, the breakthrough of immune-checkpoint inhibitors to release the brakes on the immune system clearly showed the need to identify immunogenic T-cell epitopes to use for personalized therapeutic cancer vaccines.^2^ Currently, the direct isolation of the entire Human Leucocyte Antigen (HLA)-presented peptide pool is the only reliable approach to identify the naturally presented HLA-I landscape in human cell lines,^3,4^ tumor tissues,^5−7^ and bodily fluids such as plasma.^8^ This methodology is based on immunoaffinity purification (IP) of HLA-I complexes from mild detergent-solubilized lysates, followed by extraction of HLA-I peptides. Then, the peptides are separated by chromatography and directly injected into a mass spectrometer (MS). Currently, several techniques originating from an immunoaffinity purification approach are suitable for immunopeptidomics analysis.^9,10^ Indeed, significant technological advances in chromatography, MS, and bioinformatics tools have facilitated the analysis of thousands of HLA-I peptides and enabled a greater understanding of the dynamic nature of the entire HLA-I landscape in tumor cells.

Nevertheless, in the last 20 years, few improvements related to the exploration of other methodologies have been reported in the entire immunopeptidomic pipeline, making this step open to further advancements.^11^ In particular, the limited size/amount of clinically relevant samples challenges the IP efficiency. Indeed, human informative samples (*e.g.*, tissue needle biopsies/1 mg) are smaller than the required amount of material (1 g or 1 cm^3^) to extensively study the ligandome profile of the tumor tissue.^12^ Therefore, the pooling of several samples is often required to reach a suitable amount that fits the requirements of the current immunoaffinity method, making analysis of samples from a single individual very challenging.^12^ In addition, several studies reported that IP technology is the origin of significant peptide loss during sample preparation.^13,14^ A key to achieve a comprehensive HLA peptide profiling is the development of the entire workflow including the preanalytical process prior to liquid chromatography–mass spectrometry (LC–MS)-analysis; an increased sensitivity in the methodology requires standardized protocols with comparable results between different laboratories and standardized controls.^13^ Here, we sought to establish and characterize a microfluidics-based immobilization strategy for IP of the HLA-antigen landscape for MS-based immunopeptidomics analysis that is suitable for both basic and translational studies. Specifically, we carried out the entire workflow in a single thiol–ene polymer-based microfluidic chip incorporating streptavidin-functionalized micropillars for immobilization of a biotinylated antipan-HLA antibody. By the addition of the microfabricated pillar array, the surface-to-volume ratio of the microfluidic chip could be increased by about 4-fold (compared with hollow channels) to maximize the peptide binding. The described protocol (including microchip fabrication and functionalization) can be conducted within 1 day, whereas the currently used methodologies for the antigen landscape investigation require 2 days. Thus, our technology is also somewhat faster than the traditional methods in the field.

Moreover, the cost of ligandome investigation with the conventional IP methodology is significantly high as a result of the great consumption of the affinity matrix which requires in-house production of a relatively large amount of monoclonal antibodies from hybridoma cells.^13^ Our approach integrates a miniaturized sample preparation system into immunopeptidomics analysis, leading to low reagent consumption, hence reducing the use of these expensive reagents as well. Both of these advantages, fast speed (<24 h) and miniaturization, also enable the processing of cancer patient tissue samples/*ex vivo* cell cultures that could be exploited for personalized T-cell therapies in precision cancer medicine.

In our work, we exploited the thiol–ene polymer based micropillar chip to implement the immunopeptidomics workflow, including careful analysis of the robustness of our technology and further validating it through relevant *in vitro* assays.

## Results and Discussion

### Customized Microfluidic Pillar Arrays Represent a Reliable Approach for Antibody Immobilization in Antigen Discovery Applications

We envisioned that all the immune-purification steps could be carried out within a single microfluidic chip by adding a biotinylated pan-HLA antibody to a streptavidin-prefunctionalized solid support structure (*i.e.*, the micropillar array) and eventually immobilizing the HLA-I complexes onto the pan-HLA antibody coated solid surface. In the present study, the protocol was translated to the microchip-scale to address the diverse limitations posed by the current state-of-the-art methodologies (*e.g.*, limited availability of samples, expensive consumable materials, including monoclonal antibody).

**Figure 1:**
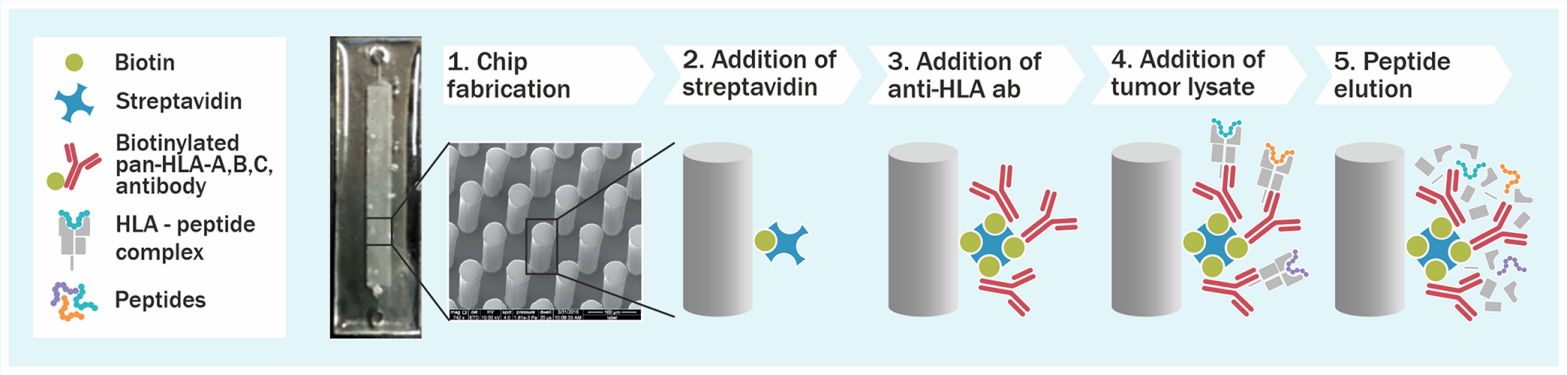
Microchip technology as an immunopurification platform for fast antigen discovery. A schematic overview describing the microchip methodology developed. Thiol–ene microchips incorporating free surface thiols are derivatized with biotin-PEG4-alkyne thiolene (stsStep 2) after which a biotinylated pan-HLA antibody is immobilized on the micropillar surface (step 3) and cell lysate is loaded into the microchip (step 4). After adequate incubation time and washing steps, the HLA molecules are eluted by adding 7% acetic acid (step 5).

The chip design used was adapted from a previous work^15^ and incorporated an array of ∼14 400 micropillars (diameter 50 μm). First, OSTE polymer based micropillar arrays were fabricated by a UV-replica molding technique and biotinylated as described in Kiiski *et al.*(16) Next, the biotinylated micropillars were functionalized with streptavidin, and the biotinylated pan-HLA antibody was added, after which the cell lysate was loaded directly into the microfluidic chip to selectively trap the HLA-I complexes. After adequate washing, the trapped HLA-I complexes were eluted at room temperature by applying 7% acetic acid (Figure 1). Thereafter, the protocol proceeded according to the standard immunopeptidomics workflow, including purification of the eluted HLA peptides with SepPac-C18 in acetonitrile and evaporating them to dryness by using vacuum centrifugation.

The microfluidic design used in this study, including the micropillar height, diameter, and density of the pillar array, was optimized in the previous work of Tähkä *et al.*(15) to maximize the total surface area (over volume), so as to maximize the amount of bound peptides per chip. On one hand, the micropillar diameter of 50 μm represents the practical minimum feasible for the replication process used in this study, whereas the layer height of 200 μm is the practical maximum. On the other hand, incorporation of the micropillar array ensures a significantly increased surface-to-volume ratio (by about 4-fold) compared with hollow microchannels of the same size, whereas the interpillar distance of 100 μm (center-to-center) facilitates proper filling by capillary forces and avoids blocking of the chip bioaggregrates and other particulate impurities (if existing in the sample).

To investigate the robustness of the microchip technology, the selectivity of each step of the microchip functionalization protocol was examined. On the basis of the previous work, the amount of streptavidin is linearly dependent on the concentration of biotin,^16^ and the amount of biotin correlates with the amount of surface thiols (∼15 ± 1 thiol groups per nm^2^ for the composition used in this study, according to Tähkä *et al.*)^15^ The efficiency of the streptavidin functionalization on prebiotinylated micropillar arrays was examined in the present work taking in account two different incubation times (15 min and 1 h), with the help of fluorescent AlexaFluor488-streptavidin. After 15 min, the streptavidin layer was already built (Supplementary Figure 2A). Moreover, to determine the effect of the streptavidin concentration on the final amount of immobilized biotinylated pan-HLA antibody, several concentrations of nonfluorescent streptavidin were tested in the presence of a fixed amount of the biotinylated pan-HLA antibody. In this case, the biotinylated pan-HLA antibody was incubated for 15 min followed by three washing steps with PBS (200 μL for each step). To assess the amount of the immobilized biotinylated pan-HLA antibody at each streptavidin concentration, a fluorescent-labeled AlexaFluor488 secondary antibody was used to quantify the immobilized biotinylated pan-HLA antibody. Interestingly, even a 10-fold increase in the streptavidin concentration did not much affect the amount of immobilized biotinylated pan-HLA antibody (Supplementary Figure 2B), which likely resulted from steric hindrances limiting the number of available streptavidin binding sites. Based on this finding, no further concentrations of streptavidin were explored, but the highest streptavidin concentration tested (0.1 mg/mL) was used in all subsequent experiments to ensure maximal binding of the biotinylated pan-HLA antibody. However, to further investigate the selectivity of the antibody binding onto the streptavidin functionalized micropillar surface, the impact of an additional coating step with bovine serum albumin (BSA) on the amount of immobilized biotinylated pan-HLA antibody was examined with a view to eliminate nonspecific interactions. To this end, the micropillar array was preconditioned with BSA (100 μg/mL in 15 mM PBS, 10 min incubation) after streptavidin functionalization, and the efficiency of subsequent binding of the biotinylated pan-HLA antibody was again determined with the help of the fluorescent-labeled secondary antibody. This procedure substantially reduced the amount of immobilized pan-HLA antibody in comparison to the nonpreconditioned surfaces (Supplementary Figure 2C) suggesting that nonspecific binding sites could be blocked with a simple BSA preincubation step. Therefore, the BSA incubation step was adapted for all further experiments.

Finally, we sought to characterize the maximum amount of immobilized biotinylated pan-HLA antibody that can be bound onto a single chip by using the optimized protocol. This was evaluated using multiple loading cycles of a fresh antibody batch of the same concentration (0.5 mg/mL) per a single microfluidic chip. In this case, the amount of the immobilized pan-HLA antibody was determined by comparing the pan-HLA antibody amount in the feed solution versus the output solution through an ELISA assay. It was observed that the amount of immobilized antibody increased almost linearly along with the number of loading cycles (Supplementary Figure 2D), allowing an accurate adjustment of the total amount of immobilized biotinylated pan-HLA antibody based on the number of loading cycles. After seven cycles, the amount of immobilized antibody reached the approximate amount of 45 μg, which suffices, at least theoretically, for the immunopeptidome investigation of scarce biological material as 10 mg of the pan-HLA (3.88 × 10^16^ molecules of antibody) is required by the state-of-the-art methodologies for the investigation of 10^9^ number of cells (Table 1).^17^

**Table 1:** Comparative Analysis between Microchip-Based IP Technology and the Standard Proceduret^a^

	total antibody consumed	antibody moles	no. of antibody molecules	total amount of cells to use
standard procedure	10 mg	64.5 nmol	3.88 × 10^16^	1 × 10^9^
microchip	45 μg	0.29 nmol	1.74 × 10^14^	4.5 × 10^6^

a The table reports the total amount of antibody coated into the microchip-based IP technology and the standard procedure. The amount of antibody molecules is calculated according to the following formula: , where mass in grams is the total antibody consumed, the molar mass is 155 000 g/mol (IgG isotype), and Avogadro’s number is 6.02 × 10^23^.

With the microchip setup, 1.74 × 10^14^ molecules of antibody could be immobilized and technically 4.5 × 10^6^ cells could be investigated. Taken together, these results clearly demonstrate the feasibility of the chip-based protocol for immobilizing the pan-HLA antibody, *via* quick biotin–streptavidin chemistry, which theoretically enables the identification of the HLA peptides from lower sample amounts compared with the current state-of-the-art protocols.

### Microchip-Based Antigen Enrichment Implemented in the Immunopeptidomics Workflow Allows the Identification of Naturally Presented HLA-I Peptides

To assess whether the developed thiol–ene microchip could be exploited as an IP platform for antigen discovery applications, we immunopurified HLA peptides from the human B-cell lymphoblastoid cell line JY. The JY line has high expression of class I HLA and is homozygous for three alleles common in the human population (HLA-A*02:01, HLA-B*07:02, and HLA-C*07:02)^18^ (Supplementary Figure 3A,B), and it has been extensively adopted for ligandome analysis, generating several publicly available ligandome repertoires.^3^ Consequently, the JY cell line was considered a suitable model for benchmarking the microchip-based IP technology.

**Figure 2:**
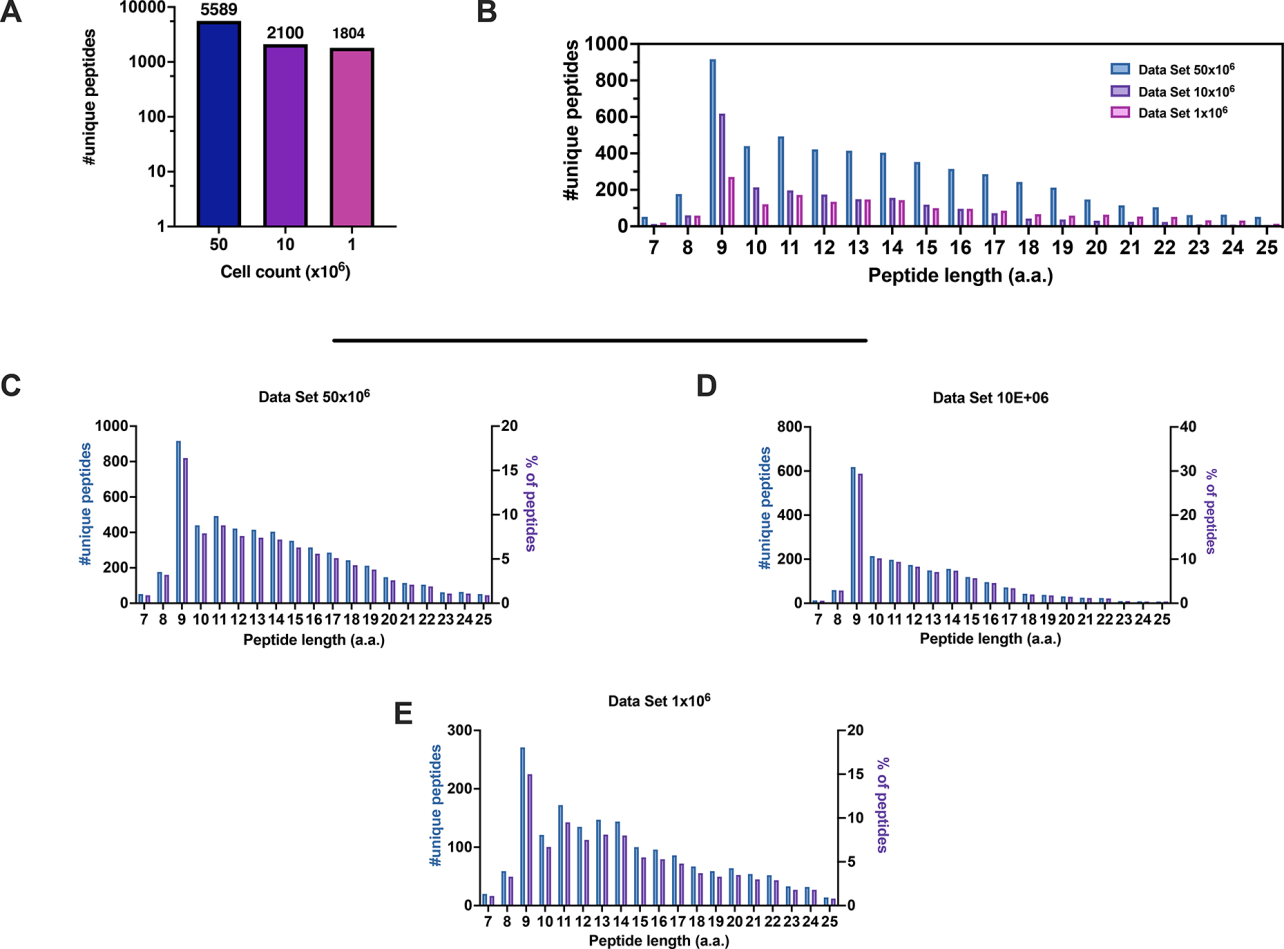
Properties of the HLA-I peptidomes data set obtained from the JY cell line. (A) Number of nonredundant peptides (unique peptides) eluted from 50 × 10^6^, 10 × 10^6^, and 1 × 10^6^ JY cells. (B) Overall peptide length distribution of the HLA peptides in the three data sets derived from the JY cell line. (C–E) Length distribution of HLA peptides is depicted as number of nonredundant (unique peptides, left *y* axis) and percentage of occurrence (right *y* axis) for 50 × 10^6^ (C), 10 × 10^6^ (D), and 1 × 10^6^ (E) cells.

Hence, HLA-I complexes were immunoaffinity-purified using the thiol–ene microchips, functionalized with the amount of pan-HLA antibody as described above. Moreover, to determine the sensitivity of our approach, the protocol was challenged by using total cell numbers as low as 50 × 10^6^, 10 × 10^6^, and 1 × 10^6^. The lysates were loaded into the microchips, and after adequate washing with PBS, the peptides were eluted with 7% acetic acid and analyzed by tandem mass spectrometry. The entire workflow took an average from the streptavidin functionalization to the elution of the tumor peptides of <24 h. A stringent false discovery rate threshold of 1% for peptide and protein identification was applied to generate data with high confidence. We were able to identify 5589, 2100, and 1804 nonredundant peptides from 50 × 10^6^, 10 × 10^6^, and 1 × 10^6^ cells, respectively (duplicates for each condition) (Figure 2A).

As we sought to carefully analyze the ability of the microchip technology to enrich for natural HLA-I binders and to avoid potential coeluting contaminants, we extensively characterized the eluted peptides. First, the eluted peptides from the JY cell line represented the typical length distribution of a ligandome data set, with 9mers as the most enriched peptide species (Figure 2B–E). Next, the predicted binding affinity for the two HLA-I alleles (HLA-A*0201 and HLA-B*0702) expressed in JY cells was determined. JY cells also have a low level of the allele HLA-C*0702, but the binding motif overlaps with the motifs of HLA-A*0201 and HLA-B*0702; hence, only these alleles were considered in the subsequent analysis.^3^

**Figure 3:**
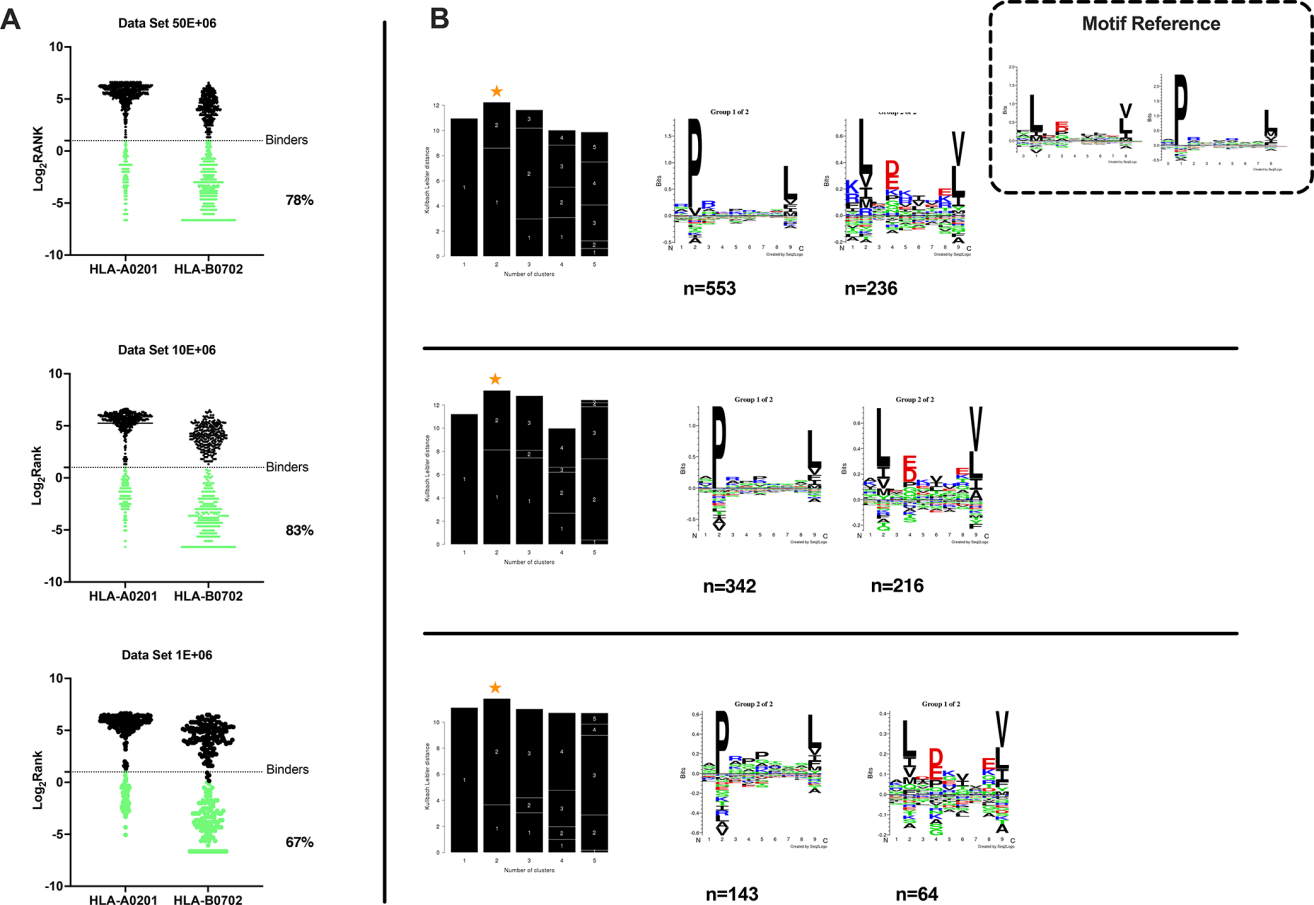
Accurate analysis of HLA ligands isolated from the JY cell line. (A) The eluted 9mers were analyzed in regards to their binding affinity to HLA-A*02:01 and HLA-B*07:02. The binders (green dots) and nonbinders (black dots) were defined in the NetMHCpan 4.0 Server (applied rank 2%). (B) HLA-I consensus binding motifs. Gibbs clustering analysis was performed to define the consensus binding motifs among the eluted 9mers peptides. The reference motif is depicted in the upper right corner. The clusters with the optimal fitness (higher KLD values, orange star) are shown, and the sequence logo is represented with the number of HLA-I for each cluster.

Of the nonredudant 9mers, 78%, 83%, and 67% were predicted to be binders (described as binders in NetMHCpan4.0, applied rank 2%)^19−21^ to either HLA-A*0201 or HLA-B*0702 alleles for 50 × 10^6^, 10 × 10^6^, and 1 × 10^6^ cells, respectively (Figure 3A). Moreover, Gibbs analysis was performed to deconvolute the consensus binding motifs of respective HLA-I alleles from the eluted 9mer peptides; these clustered in two distinct groups, with a preference for reduced amino acid complexity for residues at positions P2 and Ω, matching well with the known ones for HLA-A*0201 and HLA-B*0702 (Figure 3B).

**Figure 4:**
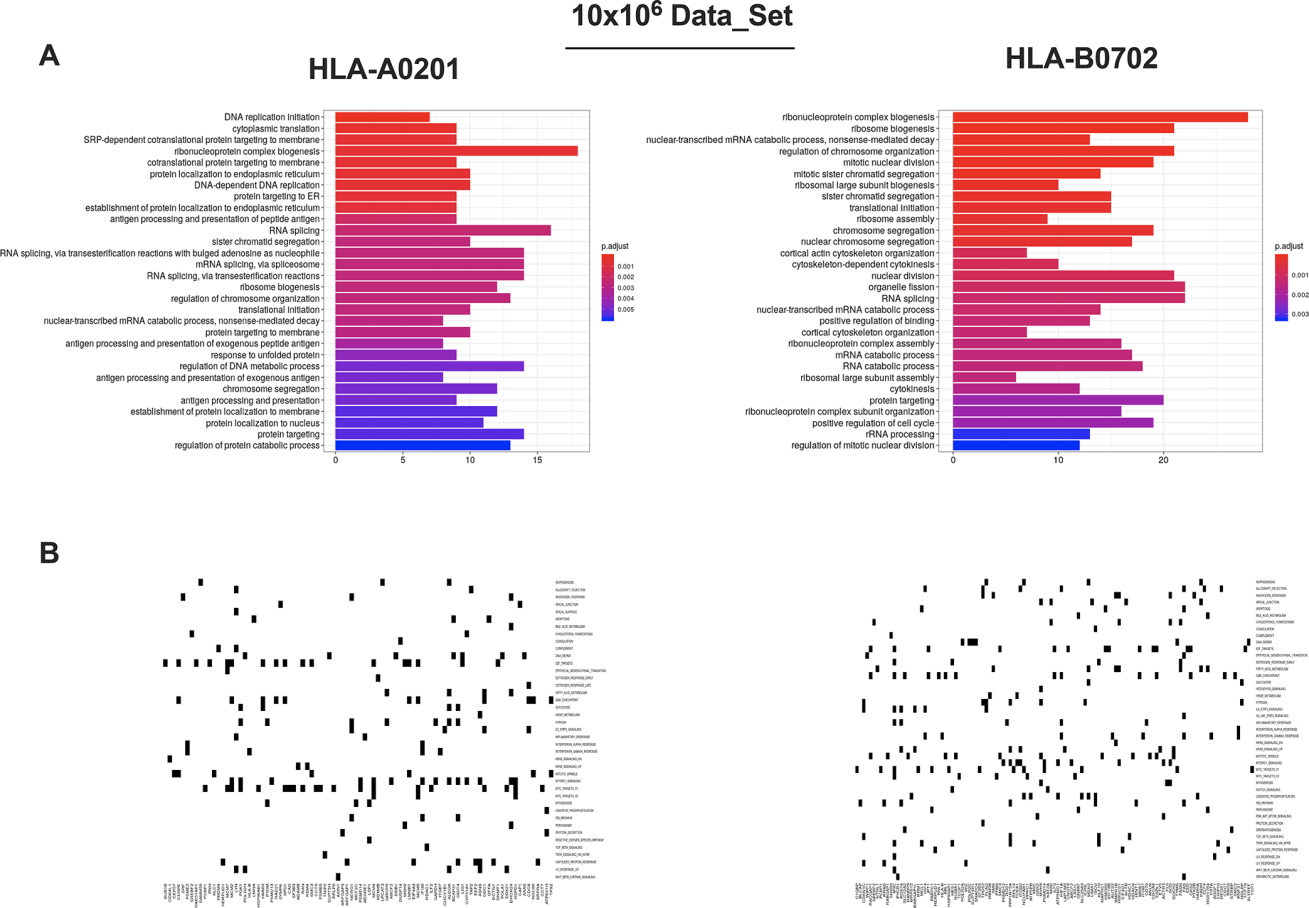
In depth enrichment analysis of HLA-ligands source proteins and CD8+ T-cell based cytotoxic assay. (A) Gene ontology enrichment analysis of the HLA-ligands source proteins. The most overrepresented biological processes for 10 × 10^6^ cells are separately shown for HLA-A*02:01 and HLA-B*07:02 alleles (hypergeometric test padj <0.01). (B) Molecular Signature Database results are displayed. The source proteins analysis was performed against the hallmark data set and separately for HLA-A*02:01 and HLA-B*07:02 alleles.

**Figure 5:**
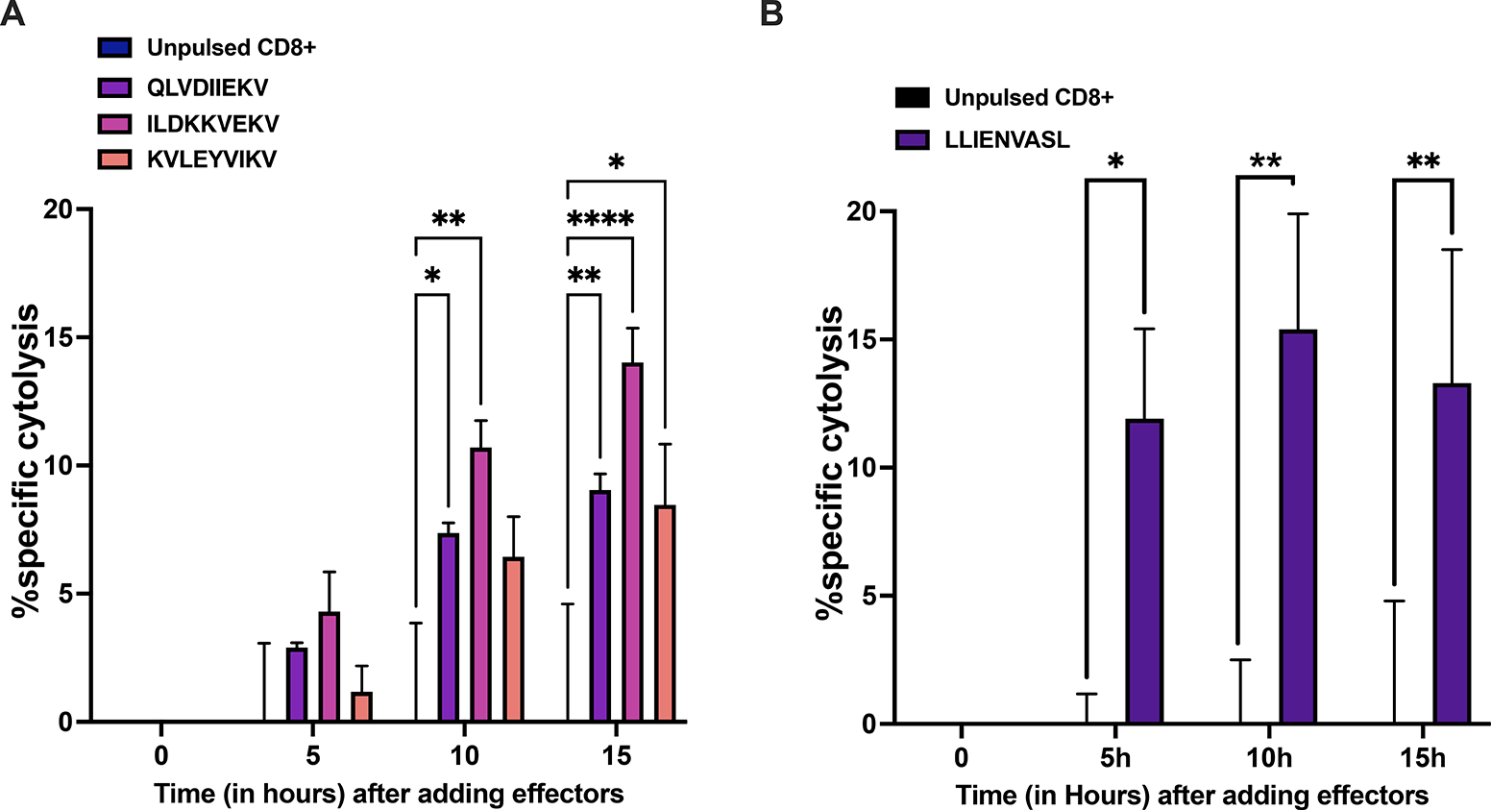
(A) PBMCs from healthy donors were pulsed for 9 days with the indicated peptides, and at day 10, CD8+T cells were isolated and used in an *in vitro* killing assay at E/T 1:1. The time-response is showed after adding the effectors. (B) PBMCs from healthy donors were pulsed for 9 days with the candidate peptide and at day 10 CD8+T cells were isolated and used in an *in vitro* killing assay at E:T 1:1. The time-response is showed after adding the effectors. The data are shown as the mean ± SEM and the significance was assessed by 2-way ANOVA, ****p < 0.0001, ***p < 0.001, **p < 0.01, *p < 0.05. The results are plotted as bar graphs (*n* = 2–4).

Next, in order to determine the role of the peptides identified, a gene ontology (GO) term enrichment analysis was performed on our list of 9mer binder source proteins. We observed an enrichment in nuclear and intracellular proteins, mainly those interacting with DNA and RNA or involved in catabolic activity (Figure 4A) (Supplementary Figure 4). These findings were in line with reports derived from other data sets.^22−24^ Intriguingly, the Molecular Signature Database (MSigDB) analysis reflected immune-associated and intracellular pathways important for B-cell biology. Indeed, we found proteins involved in IL-6 signaling (required for B-cell maturation), PI3K signaling in B lymphocytes (crucial in B-cell development), JAK2 in cytokine signaling (very active in stimulated B cells), and TGF-β signaling (regulator of B cell development and function) (Figure 4B) (Supplementary Figure 5).^25,26^ Finally, we set up an *in vitro* killing assay to further demonstrate the capacity of the microchip technology in isolating peptides in complex with HLA-I. To this end, a set of three peptides was selected from our JY data set to stimulate HLA-matched PBMCs; CD8+ T cells were purified from the PBMCs and adopted as effector cells in the coculture with JY cells. To account for nonspecific cytotoxicity due to the effector cells *per se*, unstimulated PBMCs were used as a control. Real time cytolysis was then monitored. Interestingly, the CD8+ T cells pulsed with the peptides QLVDIIEKV (gene name PSME3) and KVLEYVIKV (gene name MAGEA1) showed ∼10% specific cytolysis, whereas the CD8+ T cells pulsed with the peptide ILDKKVEKV (gene name HSP90AB3P) induced 15% specific cytolysis (Figure 5A), indicating specific lysis in the presence of defined peptides. Next, we sought to investigate whether the microchip technology could identify peptides to exploit as cancer immunotherapeutic targets. To this end, the list of peptides was analyzed through HEX software; this latter is a tool previously developed by Chiaro *et al.*(4) that is able to identify tumor antigens similar to pathogen antigens in order to exploit molecular mimicry and tumor pathogen cross-reactive T-cells in cancer vaccine development.^27,28^ We focused our attention on the peptide LLIENVASL (gene name GPX1) identified as identical to one peptide derived from the virus Molluscum Contagiosum. Moreover, this peptide has been described as MHC-I ligands also by other groups (IEDB database); however, no T cell assays have ever been performed to validate it as a possible target for immunotherapy. Therefore, we purified CD8+ T cells from the peptide pulsed PBMCs, and we used them as effector cells in coculture with JY cells. To account for nonspecific cytotoxicity due to the effector cells per se, unstimulated PBMCs (unpulsed CD8+) were used as a control. Real time cytolysis was then monitored, and specific cytolysis was calculated. The peptide LLIENVASL was able to induce ∼15% specific cytolysis (Figure 5B), confirming this peptide as a possible immunotherapeutic target.

**Figure 6:**
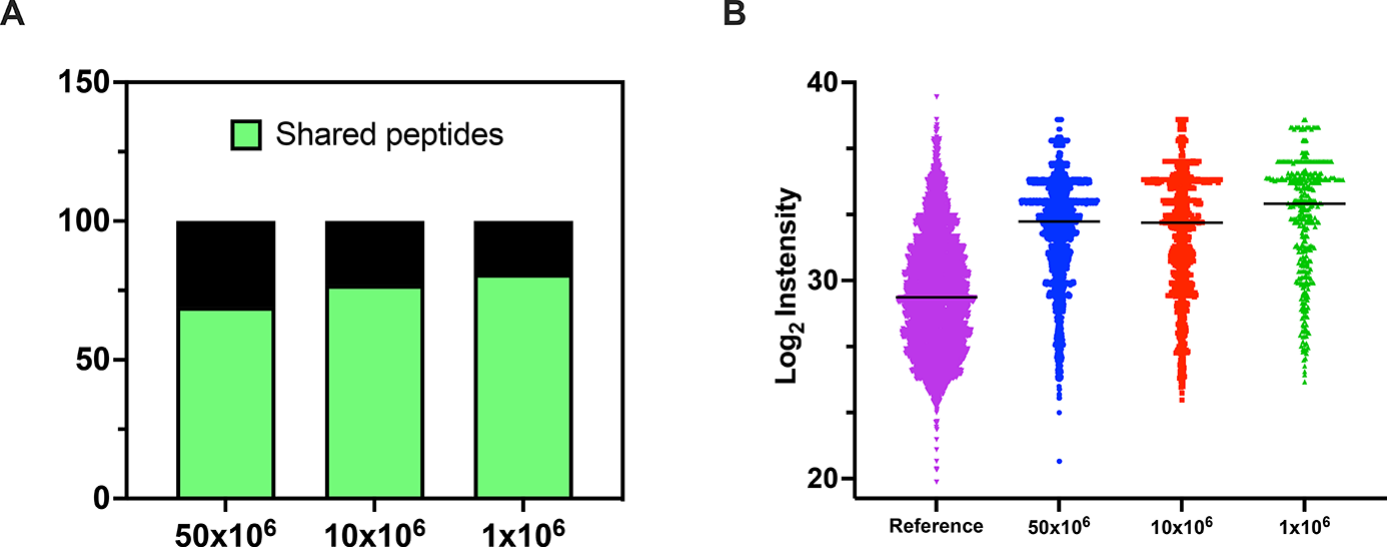
Comparative analysis of the generated data sets from JY cell line. (A) The percentage of the shared 9mers has been calculated against a depository reference data set (pride ID PXD000394) derived from the JY cell line; the results are depicted as a bar plot, and the percentage of shared peptides is indicated in green. (B) The abundance of the source proteins is expressed in log_2_ intensity, and the values are derived from a reference published proteomic analysis of the total JY cell lysate. The plot showed the comparison among the three data sets (50 × 10^6^ cells, 10 × 10^6^ cells, and 1 × 10^6^) generated through our microchip technology and the reference data set.

To evaluate the validity of our HLA-I peptide lists identified by the microchip technology, we interrogated SysteMHC, a repository of the immunopetidomics data set generated by mass spectrometry. Among the nonredundant 9mer binders identified in our data, 69%, 77%, and 81% were also found in a previously published ligandome data set derived from the JY cell line (pride ID PXD000394)^3^ for 50 × 10^6^, 10 × 10^6^, and 1 × 10^6^ cells, respectively (Figure 6A). In addition, a positive correlation between the abundance of the source protein and HLA presentation has been previously reported.^3^ Here, we sought to determine whether the same tendency was confirmed in our data sets. To this end, previously published JY proteomics data^3^ were used to retrieve the log_2_ intensity of the source proteins present in our data sets. The analysis confirmed the previous assumption, with the most abundant proteins being the main source of the HLA peptides (Figure 6B).

Furthermore, six peptides have recently been reported as natural HLA-I peptides from JY cells and have been used by Ghosh *et al.*(29) to validate immunopeptidomic assays suitable for pharmaceutical therapies. In line with this previous observation, some of these peptides were also found in our data sets from 50 × 10^6^ cells (three peptides), 10 × 10^6^ cells (four peptides), and 1 × 10^6^ cells (three peptides) (Table 2). Finally, we further investigated side by side the ligandome output profile derived both from the standard method and the microchip at two different numbers of cells, 1 × 10^6^ and 10 × 10^6^.

**Table 2:** Natural HLA-I Peptides Isolated in JY Cell Line Validated Immunopetidomic Assay^a^

naturally HLA-I peptides (Gosh *et al.*)	50 × 10^6^	10 × 10^6^	1 × 10^6^
AIVDKVPSV		√	√
RPSGPGPEL	√	√	√
YLLPAIVHI			
KVLEYVIKV	√	√	
SPSSILSTL			
SPQGRVMTI	√	√	√

a The table depicts on the left the list of naturally HLA-I peptides used to validate immunopeptidomic assay suitable for pharmaceutical therapies. On the right is the list of the peptides found in our data sets for 50 × 10^6^, 10 × 10^6^, and 1 × 10^6^ cells indicated as a check mark.

Across two biological replicates, a total of 1134 and 387 nonredundant peptides were identified, respectively, for 10 × 10^6^ and 1 × 10^6^ cells by the standard method (Supplementary Figure 6). In contrast, in regards to the number of nonredundant peptides, the microchip isolated 2100 from 10 × 10^6^ cells and 1804 from 1 × 10^6^ cells (Supplementary Figure 6). Therefore, the microchip showed higher detection sensitivity at the lowest amount of sample.

**Figure 7:**
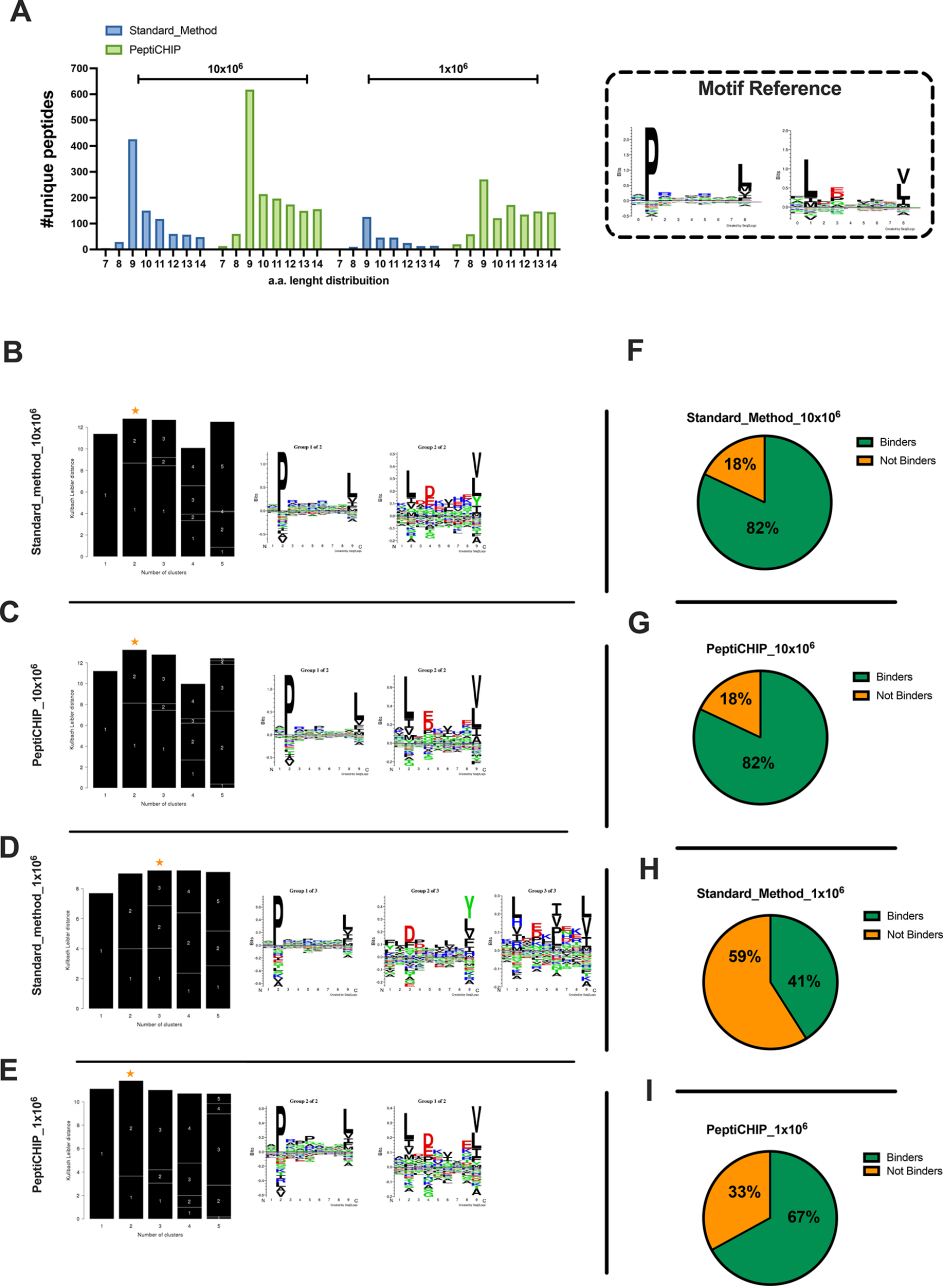
Comparative analysis between the standard method and PeptiCHIP technology. (A) Peptide length distribution is reported for the standard method (blue bars) and the microchip (green bars) for each number of cells (left, 1 × 10^6^ cells; right, 10 × 10^6^ cells). (B–E) The 9mers consensus binding motifs were deconvoluted by unsupervised Gibbs clustering analysis for both the standard method and the microchip and for each number of cells. The reference motif is shown in the upper right corner. The clusters with higher KLD values were chosen (orange star), and the sequence logo is reported. (F–I) The binding affinities to HLA-A*02:01 and HLA-B*07:02 were predicted for the eluted 9mers, and the percentages of binders and not binders are depicted as a part to the whole for both the standard method and the microchip and for both numbers of cells.

Next, the eluted peptides showed the typical ligandome length distribution with the 9mers as the most enriched species in both the standard method and the microchip, depicting the same tendency in retrieving a higher number of peptides from 10 × 10^6^ compared to 1 × 10^6^, well in line with the decreasing availability of HLA-complexes due to diverse number of cells (Figure 7A). Next, unsupervised Gibbs clustering analysis was performed to deconvolute the consensus binding motifs of respective HLA-I alleles from 9mer peptides. At 10 × 10^6^ cells, in both approaches the 9mers clustered in two groups, clearly resembling the HLA-A*0201 and HLA-B*0702 reference binding motifs (Figure 7B,C); however, at the lowest number of cells (1 × 10^6^), the 9mers derived from the standard method clustered in three groups (Figure 7D), highlighting the presence of contaminants; instead, the microchip at 1 × 10^6^ cells was still able to isolate peptides that clustered in two distinct groups, confirming a better efficiency in the immunoaffinity purification at small amount of cells (Figure 7E). Moreover, for 10 × 10^6^ cells, among the nonredundant peptides, 82% of 9mers Figure 7F,G were predicted to be binders (described as binders in NetMHCpan4.0, applied rank 2%) to at least one allele in both the standard approach and the microchip. Strictly, the number of binders dropped to 41% in the standard method (Figure 7H) in contrast to 67% for the microchip output (Figure 7I), confirming that at the lowest amount of sample, the microchip showed a better efficiency. Hence, these results demonstrated that the chip-based protocol can be exploited as a reliable IP platform within the immunopeptidomic workflow, providing a potential alternative to the current state-of-the-art technology.

### The Microfluidic Chip-Based Platform Investigates the Immunopeptidome Profile in Scarce Tumor Biopsy Tissue

**Figure 8:**
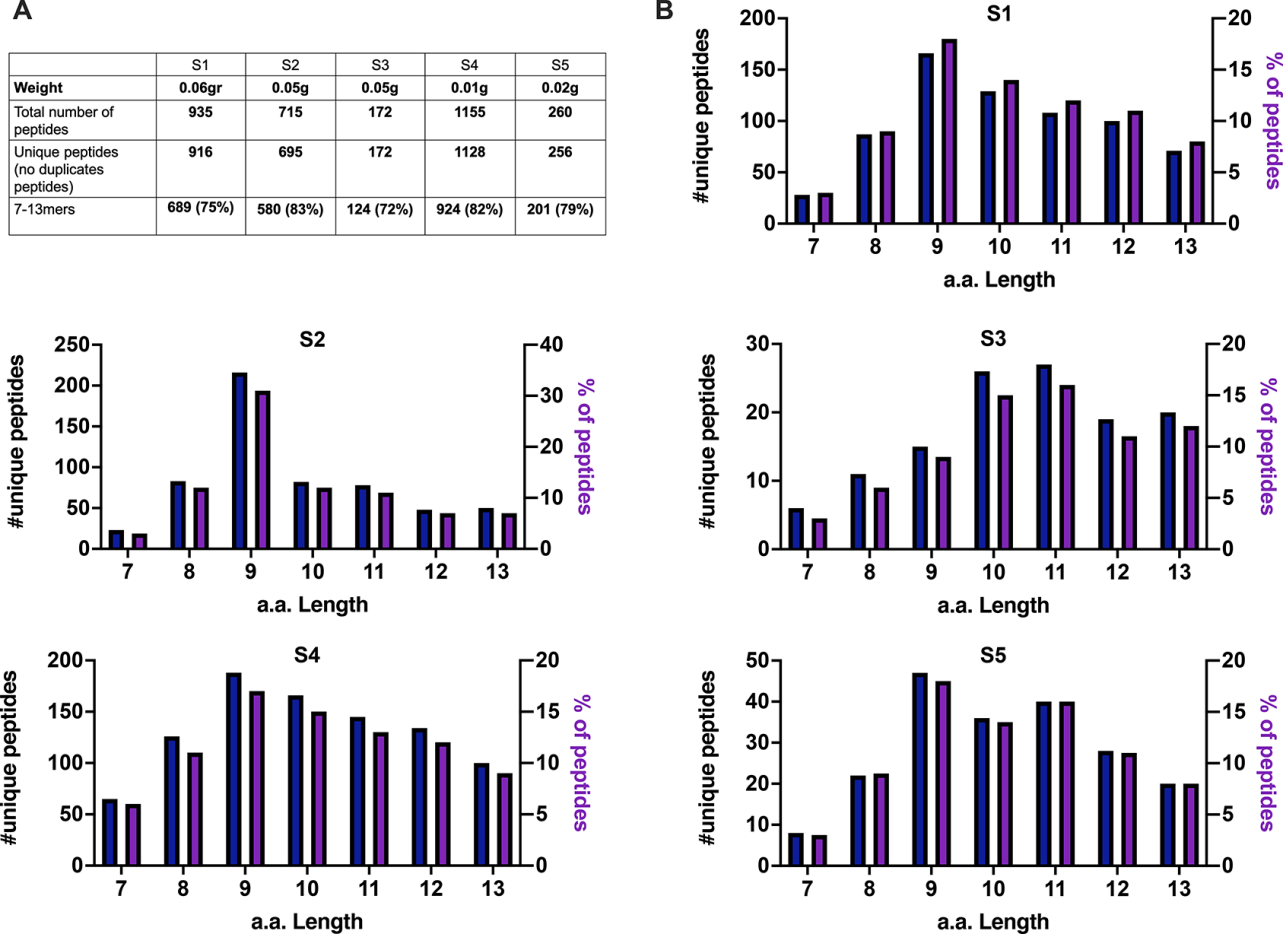
Microchip based platform reveals the immunopepetidomic profile in scarce tumor biopsies. (A) The weight of the samples before the processing, the total number, and the nonredundant (unique peptides) and the enrichment in 7–13mers specimens are summarized here. (B) The length distribution of the peptides in regards to their absolute number and the percentage are shown as bar plots.

As we demonstrated that the microchip-based technology can be exploited for the ligandome analysis of the model cell line JY, we aimed to challenge the platform for the investigation of a scarce tumor biopsy. Thus, an ovarian metastatic tumor (high grade serous) was collected from the patient, and four pieces were derived from the tumoral border (S1, S2, S3, and S4); the central part of the tumor was collected as well (S5). Next, the samples were weighed, and as summarized in Figure 8A, the size averaged from 0.01 g to 0.06 g. After the sample digestion, the obtained single cells suspension was lysed and processed through the microchip. Applying a stringent false discovery rate threshold of 1% for peptide and protein identification, 916, 695, 172, 1128, and 256 nonredundant peptides were identified, respectively, in S1, S2, S3, S4, and S5 (Figure 8A). In line with a typical ligandome profile, a general enrichment (above 70%) was observed in 7–13mers specimens (Figure 8A). In regards to the absolute number and percentage, the amino acidic length distribution showed that the 9mers specimens were the most representative (Figure 8B), confirming our and other previous immunopeptidomic analyses. Next, we further investigated the source proteins found in our data, applying Gene Ontology (GO) enrichment analysis. Consistently with a typical ligandome profile, metabolic processes were enriched in all the samples examined (Supplementary Figure 7A). Additionally, the analysis revealed an increase in skin the development pathway in line with the epithelial nature of the ovarian serous tumor analyzed here. Altogether, the results highlighted the feasibility of exploiting the developed microfluidic-chip platform to analyze a scarce tumor biopsy.

### The Microchip-Based Protocol Reveals the Immunopeptidome Landscape in Patient-Derived Organoids

After demonstrating the feasibility of microchip-based IP technology for ligandome discovery, we sought to determine whether this technology could be applied to investigate the immunopeptidome landscape of scarce patient-derived clinical material. To this end, the microchip technology was challenged with as few as 6 × 10^6^ cells from patient-derived organoids (PDOs). We selected two patients from an ongoing precision medicine study for urological cancers, a nephrectomy sample containing both benign and cancer tissues from a clear cell renal cell carcinoma (ccRCC) patient, and a 1 × 1 cm sample from a bladder cancer patient, which were further processed as 3D primary organoid cultures.

**Figure 9:**
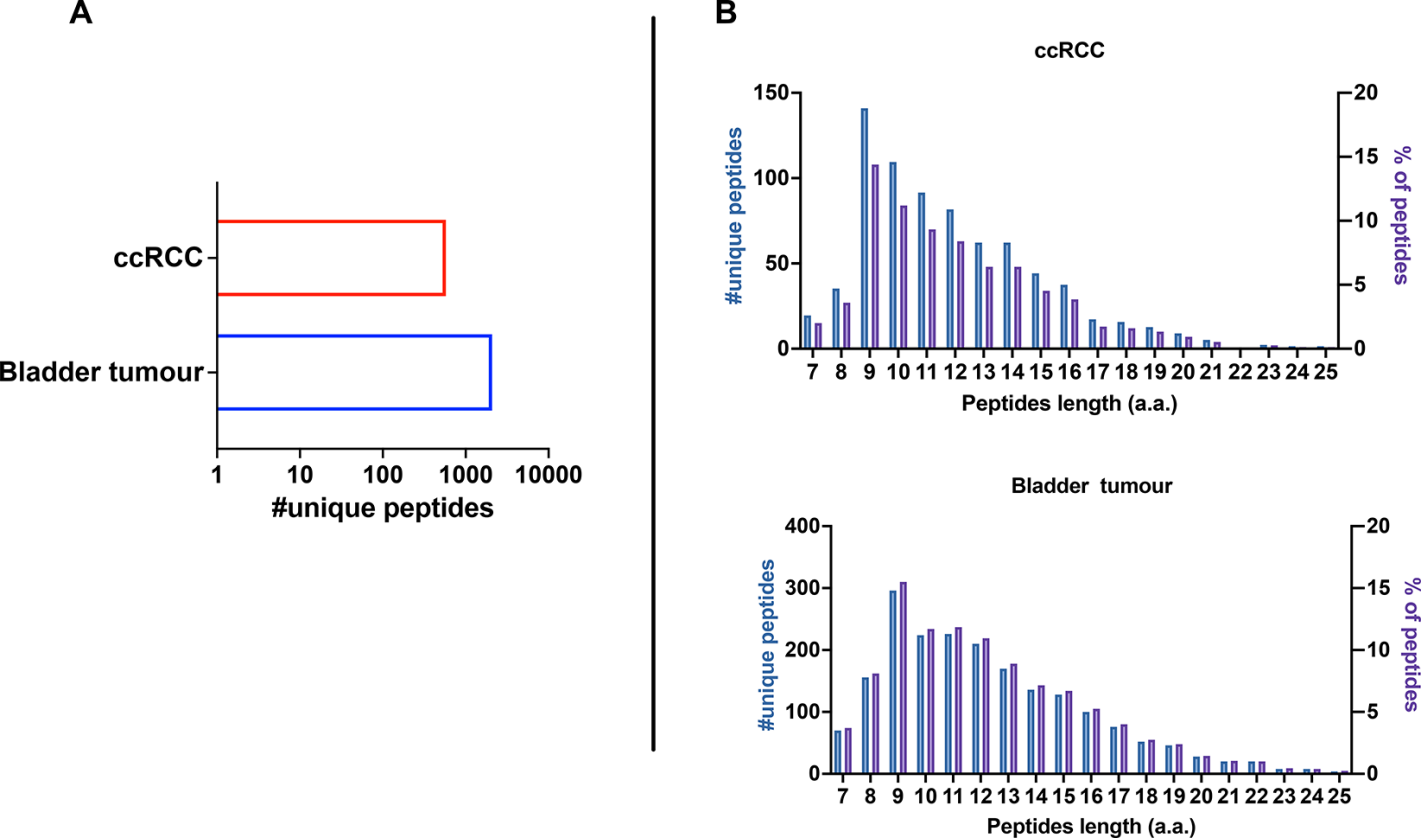
Immunopeptidomic analysis of ccRCC and Bladder tumor patient derived organoids (PDO). (A) Number of nonredundant (unique peptides) detected in ccRCC and bladder PDOs. (B) The peptides length distribution is shown as the total number of nonredundant peptides (unique peptides, left *y* axis) and percentage of occurrence (right *y* axis) per each PDO (ccRCC, upper panel; bladder, lower panel).

**Figure 10:**
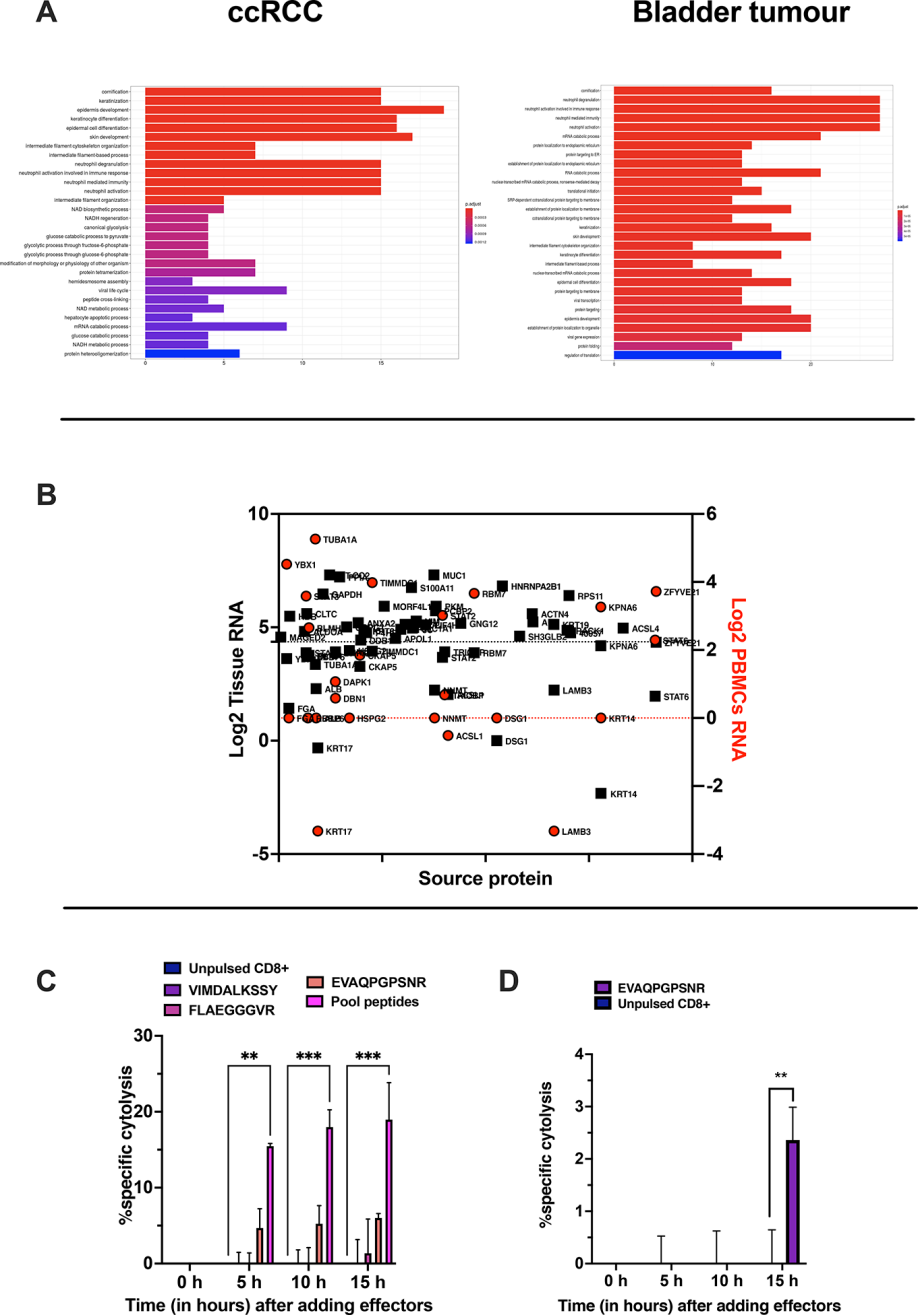
Assessment of the immunopeptidomic profile in PDOs. (A) Gene ontology enrichment analysis. The most overrepresented biological processes for RCC (left panel) and bladder (right panel) PDOs are shown (hypergeometric test padj <0.01) (B) The source proteins expression is depicted as log_2_ of the RNA level in healthy kidney tissue (black square) and in blood PBMCs (red circle). The 1st quartile is indicated as black and red dashed line, respectively, for the healthy kidney tissue and blood PBMCs. (C) PBMCs from healthy donors or (D) PBMCs from a patient were pulsed for 9 days with the indicated peptides, and at day 10 CD8+T cells were isolated and used in an *in vitro* killing assay at E/T 1:1. The time-response is shown after adding the effectors. The data are shown as the mean ± SEM, and the significance was assessed by 2-way ANOVA, ****p < 0.0001, ***p < 0.001, **p < 0.01, *p < 0.05. The results are plotted as bar graph (*n* = 2).

Applying the developed microchip technology and a stringent false discovery rate threshold of 1% for peptide and protein identification, we were able to identify a total of 576 and 2089 nonredundant peptides in ccRCC and bladder PDOs, respectively (duplicates for each PDO) (Figure 9A). The number of retrieved peptides differed between the two samples, with bladder PDOs resulting in more peptides than ccRCC PDOs. It is well-known that HLA expression influences the amount of isolated HLA-I peptides,^17^ and consistent with this, flow cytometry analysis revealed higher surface levels of HLA-A, HLA-B, and HLA-C in our bladder PDOs than in our ccRCC PDOs (Supplementary Figure 8A,B), explaining the different yields of retrieved peptides from our samples. Analysis of the peptides showed a preference for 9- to 12mers (56.4% in ccRCC and 47.9% in bladder tumors) with an enrichment in the 9mer population, in line with the typical length distribution of ligandome analysis (Figure 9B).^30^ To discriminate between HLA-I binders and contaminants, Gibbs clustering and NetMHC4.0 analysis was performed. First, deconvolution of the 9mers showed that 55% and 67% in bladder and ccRCC PDOs, respectively, matched at least one of the patients’ HLA alleles (Supplementary Figure 9A,B). Next, NetMHC4.0 was applied to all 9mers identified in our data set. Among them, 46% and 69% were predicted to be binders of the specific HLA of the patients (Supplementary Figure 9C). Next, the source proteins present in both our data sets were investigated. To this end, Gene Ontology (GO) enrichment analysis was performed. Consistent with our previous observations and published data sets,^22−24^ both samples showed an enrichment in intracellular and nuclear proteins interacting with RNA and involved in catabolic/metabolic processes. Interestingly, the analysis highlighted the enrichment of biological pathways crucial for neutrophil activity (Figure 10A), confirming the immune-infiltrated nature of the tumor types.^31^

To demonstrate that the microchip technology can be exploited for the rapid development of therapeutic cancer vaccines, we decided to set up a killing assay using PDOs as targets and PBMCs pulsed with the identified peptides as effector cells. As the amount of bladder PDOs was insufficient to proceed with further *in vitro* validation, we focused our analysis on ccRCC PDOs on the RNA level of the source proteins contained in our data set. We used transcriptomics levels to select putative tumor antigens using PBMC and healthy kidney tissue as reference sets (RNA data were retrieved from The Human Protein Atlas,^32^ and the first quartile of Log2 RNA was considered) (Figure 10B) Next, PBMCs from healthy volunteers were pulsed with the selected peptides, and CD8+ T cells isolated from those cells were used in the assay. T cells pulsed with the peptide EVAQPGPSNR (gene name HSPG2) showed ∼10% specific cytolysis (Figure 10C); in contrast, the peptides VIMDALKSSY (gene name NNMT) and FLAEGGGVR (gene name FGA) were ineffective in eliciting specific CD8+ T cell responses. Interestingly, the pool of peptides (EVAQPGPSNR, VIMDALKSSY, and FLAEGGGVR) reached a specific cytolysis of 15% (Figure 10C) in our assay, overcoming the limits of the single peptide specific CD8+ T cells. Finally, we sought to investigate the recall T cells response in the ccRCC patient. To this end, unfractionated PBMCs derived from the patient were *in vitro* stimulated with the peptide EVAQPGPSNR, whereas unstimulated PBMCs were adopted as a control. The derived CD8+T cells were then added to the ccRCC PDO showing an increased killing activity compared to the control group (Figure 10D).

Overall, these data support the potential application of the developed microchip technology with limited clinical samples, overcoming the limitations encountered in ligandome discovery for tumor biopsies.

The breakthrough of immune-checkpoint inhibitors (ICIs) targeting PD-1, PD-L1, and CTLA-4 and their clinical approval^33^ have attracted increasing interest in the cancer immunotherapy field. Despite their clinical success, objective response rates are not yet satisfactory (20–30% for many types of cancer),^34^ highlighting the need to combine ICI with approaches that aim to generate and sustain specific antitumor CD8+ T cells (*e.g*., therapeutic cancer vaccines).^35,36^ In this scenario, to design effective tumor rejection and protection strategies, the reliable identification of tumor peptides binding to HLA-I has become a hot topic.^6,37,38^ The direct identification of the peptides from the HLA-I complex still represents the best-established and the most widely used method for their identification.^39^ Nevertheless, the immunopeptidomics workflow is relatively complex and thus represents a limitation in the antigen discovery process.^11^ Currently, the inability to analyze immunopeptidomes from a small amount of biological materials (*e.g.*, tissue needle biopsy), the sample throughput and the heavy antibody consumption in the current IP platform have been depicted as the main technical challenges to address.^12^ Moreover, the Human Immuno-Peptidome Project (HIPP) meeting report indicated that during the purification steps, only approximately 0.5–3% of HLA peptides are recovered, with most peptides lost during IP,^13,14^ making this procedure the major technology gap in the overall workflow.^40^

It is clear that we need to explore and develop strategies for the isolation of HLA-I peptides and that the field would greatly benefit from additional technical advancements.^13^ In this work, we proposed a possible solution to overcome several technical issues hindering the ligandome research, with the main focus on the limited availability of material to analyze, on the cost of consumables, and on prolonged protocols.

By exploiting the well-characterized biotin–streptavidin interaction to immobilize biotinylated pan-HLA antibody on streptavidin-functionalized surfaces, we were able to replace the current technology based on affinity matrices prepared *via* cross-linking reactions with a microchip platform, reducing time and cost. Indeed, our microchip requires only 45 min to generate an immunoaffinity matrix, whereas the standard approach demands 2 h and 20 min.^17^ Moreover, the current HLA-immunoprecipitation spans for 3 h and 30 min; in contrast in our technology, the addition of the cell lysate overnight is followed by 5 min elution on the following day.^17^ The limitations posed by the paucity of the material (*e.g*., needle biopsy) inspired the work toward the implementation of a microfluidic protocol. In this work, we employed a custom microchip protocol involving a thiol–ene polymer-based micropillar array as the solid support for further biofunctionalization that enabled performing the entire IP procedure on a single microfluidic chip.

Thiol–enes are an emerging class of polymers that facilitate not only low-cost microfabrication *via* noncleanroom replica molding but also implementation of a wealth of subsequent, tailor-made surface functionalizations at a significantly lower cost.^41,42^ As our approach offers an unexplored tool for IP, the custom-designed protocol necessitated careful analysis of its specificity and robustness. Therefore, several characterization steps and thorough method validation were performed to establish the technical basis of the protocol.

First, every step of microchip surface functionalization was examined to set up the best experimental conditions for subsequent steps. By incorporating the microfabricated pillar architecture, the total surface area (*A*) to sample volume (*V*) ratio could be increased by about 4-fold from ∼*A*/*V* = 10.7 mm^–1^ for “hollow” microchannels of the same size to ∼*A*/*V* = 39.0 mm^–1^ to micropillar arrays. This was critical to increasing the binding capacity while ensuring flawless filling of the chip thanks to the well-defined micropillar array. Instead of coating the microchip surface directly with the biotinylated pan-HLA antibody, we decided to build the first layer with streptavidin bound onto a prebiotinylated (with biotin-PEG4-alkyne) microchip surface so as to get a longer linker out from the pillar surface, enhancing the antibody binding capacity. The effect of the streptavidin concentration was analyzed in regard to its binding efficiency and then to the amount of immobilized antibody, which was shown to increase in a streptavidin concentration-dependent manner. However, even 10-fold increases in the tested concentrations showed only small differences, suggesting that the saturation of the antibody binding capacity was achieved. To further increase the selectivity of the interaction between streptavidin and the biotinylated pan-HLA antibody, we also included BSA incubation as the blocker step, after streptavidin coating, to avoid nonspecific antibody interaction that could interfere with the antibody activity. As expected, BSA preconditioning decreased the total amount of bound antibody, confirming that addition of BSA as the blocking agent reduces the nonspecific interaction of the antibody with the chip surface increasing the selectivity of the binding between streptavidin and biotinylated pan-HLA antibody. Using this functionalization protocol, the amount of pan-HLA antibody immobilized onto the chip was shown to increase somewhat linearly along with antibody loading cycles so that an antibody amount of 45 μg was reached after seven cycles. This amount of pan-HLA antibody theoretically suffices for carrying out IP of scarce biological material while being substantially lower than the amount employed in the current IP platform, which is typically between 1 and 10 mg of antibody.^17^ Next, we sought to investigate the feasibility of the microchip technology for incorporation as an integral component for IP in the ligandome protocol to investigate scarce biological material. On the basis of publicly available ligandome data and on the alleles profile (the cell line is homozygous for HLA-A*02:01, HLA-B*07:02, and HLA-C*07:02), the EBV-transformed human B-cell line JY appeared as a reliable model to evaluate whether the pan-HLA antibody functionalized microchip could serve as an IP platform in the ligandome analysis.^18^

Hence, the validation of the microchip-based protocol was conducted by using the JY cell line and challenged with as few as 50 × 10^6^, 10 × 10^6^, and 1 × 10^6^ cells instead of the (5 × 10^8^) to (1 × 10^9^) cells commonly required in the current state-of-the-art IP methodologies.^17^

The peptides trapped by and eluted from the microchip clearly showed the typical length distribution of a ligandome data set, with an enrichment in 9mer species; moreover, NetMHC 4.0 analysis for HLA-A*02:01 and HLA-B*07:02 identified 78%, 83%, and 67% of the 9mers as binders in the data set derived from 50 × 10^6^, 10 × 10^6^, and 1 × 10^6^ cells, respectively. The data were further corroborated by the deconvolution analysis (unsupervised Gibbs clustering) that identified motifs resembling the reference ones. We are aware that the JY cell line also expresses a low level of HLA-Cw* 07:02; however, the binding motifs overlap with the motifs of HLA-A*02:01 and HLA-B*07:02, hindering a reliable analysis for HLA-Cw*07:02, as reported in Bassani *et al.*(3) Accordingly, our analysis was focused on the HLA-A*02:01 and HLA-B*07:02 alleles. Following these first preliminary results, we further characterized the peptide list by GO and MSigDB analyses. These latter revealed an enrichment in nuclear and intracellular proteins, in line with published ligandome data sets;^22−24^ most importantly, an enrichment in pathways essential for B-cell biology was observed, consistent with the nature of the model used in the microchip methodology (EBV-transformed human B-cell line JY). Last, the validation of the identified peptides in an *in vitro* killing assay confirm that the peptides were actually presented on the JY cell surface, as they were killed in a specific CD8+ T cell-dependent fashion. The peptide ILDKKVEKV found in our data set elicited the higher percentage of specific cytolysis. This peptide is a known B cells epitope in the Immune Epitope Database (IEDB) and interestingly it derives from the pseudogene HSP90AB3P. In line with this, altered pseudogene expression in cancer has been reported.^43^ The upregulation of peptides derived from the pseudogene could break the T cell tolerance, inducing the activation of autoreactive T cells.^44^ In this scenario, peptides from the pseudogene are an interesting target to exploit for cancer therapeutic approaches. To benchmark our results with the state of the art in the ligandome field, we carefully compared our data with well-established and solid data sets. We investigated two main factors: the presence of our eluted 9mers in the reference database and the intensity of their source proteins; as a result, an average of 76% of the nonredundant 9mers were also found in the reference database, and the abundance of the source proteins clearly showed a direct correlation with HLA presentation, consistent with previous observations.^3^ Moreover, the six peptides (AIVDKVPSV, SPQGRVMTI, RPSGPGPEL, YLLPAIVHI, KVLEYVIKV, and SPSSILSTL) recently listed as natural HLA-I peptides from the JY cell line in Ghosh *et al.*(29) were also present in our data sets. The comprehensive technical characterization and the method validation results derived from the microchip-based protocol in ligandome analysis of the model JY cell line clearly evidenced that the microchip protocol was a robust tool to be integrated with the immunopetidomics workflow and that the methodology could be exploited to investigate the antigen landscape of scarce clinically relevant material. This is a key aspect for applicability, since the primary target population, metastatic cancer patients, rarely undergo operations and therefore samples are mainly obtained by needle biopsies. The number of cells derived from a needle biopsy account from (1.65 × 10^6^) to (6 × 10^6^), with differences depending on the tumor models and medical personnel expertise.^45^ In the clinical setting, the patient sample size often hinders HLA peptidome discovery, and several attempts in the field have been attempted in the field to tackle this limitation, for instance, by establishing cell lines or the use of patient-derived xenograft mouse models.^18,41^ However, the manipulation of the patient-derived samples to obtain a sufficient amount of biomaterial is time- and cost-intensive, and additionally, it could compromise the biological significance. To assess whether the microchip technology could address these issues at least in part, we applied the microchip technology for ligandome analysis of scarce ovarian tumor biopsies and then for the analysis of ccRCC and bladder tumor PDOs. The microchip-based technology was successfully exploited for the ligandome analysis of multiple scarce ovarian tumor biopsies, investigating the ligandome profile of samples inaccessible to state-of-the-art methodologies in the field.

Recently, PDO cell pellets (biological replicate, (3.85 × 10^7^) to (1 × 10^8^) cells/pellet) from colorectal cancer have been extensively investigated in immunopeptidomic analyses.^46^ In this work, we challenged the microchip technology by scaling down the amount of PDO cell pellets down to 6 × 10^6^ cells for each sample. As expected, the microchip was able to isolate peptides that resembled the typical ligandome length distribution. Additionally, the number of eluted peptides directly relied on the HLA surface level in the PDOs, confirming that the peptides were most likely HLA ligands. Of note, the biological pathway analysis of the source proteins strongly suggested tumor immune infiltration. Indeed, ccRCC exhibits recruitment of neutrophils that in turn support cell invasion by modulating the ERβ, VEGFa, and HIF2α signaling pathways.^31^ Although bladder tissue under physiological conditions lacks neutrophil infiltration, tumoral transformation correlates with higher recruitment of neutrophils in the tumor site.^47^

Last, we validated the eluted peptides from the ccRCC PDOs in an *in vitro* killing assay. Peptide selection is of utmost importance for T cell-based cancer immunotherapies, and several strategies have been pursued so far.^40^ In this work, we selected peptides based on the RNA expression level (data retrieved from Human Protein Atlas), prioritizing peptides that retained low expression in both healthy renal tissue and PBMCs as severe or lethal side effects due to the lack of tumor specificity have been reported in several cancer vaccine approaches.^40^ This strategy allowed the selection of three peptides to be employed for the stimulation of PBMCs from healthy donor. Interestingly, only CD8+ T cells isolated from PBMCs stimulated with the peptide EVAQPGPSNR elicited specific cytolysis. This peptide derives from the heparan sulfate proteoglycan (HSPG2) and has been reported as over-represented peptides HLA-I peptide in colorectal cancer,^48^ making it a tumor associated antigen to further investigate.

## Conclusions

This work integrates chip microfluidic technologies as a component with the immunopeptidomics workflow, addressing the main issues that are universally recognized challenges in the field with regard to the scarcity of biological material, costs, long and laborious protocols, and the need for extensive sample handling. In particular, a typical ligandome experiment requires 5 × 10^8^ number of cells,^17^ whereas in this work we showed that the microchip could isolate HLA-peptides from 1 × 10^6^ cells, drastically reducing the amount of input material. From a clinical point of view, our technology pays the way to the ligandome analysis of a needle biopsy that usually accounts for (1.65 × 10^6^) to (6 × 10^6^) number of cells.^45^ Besides technical characterization and method validation, the microchip technology was adopted to the antigen discovery process of clinical samples (PDOs); among the reported results, the key finding was that our customized-designated microchip protocol was able to isolate HLA-relevant ligands from as few as 6 × 10^6^ cells instead of (3.85 × 10^7^) to (1 × 10^8^) cells, which is the state of the art as recently reported in the same context.^46^ We envision that this technology may be further developed for clinical practice in therapeutic cancer vaccine development.

## Methods and Experimental Details

### Microchip Design, Fabrication, and Functionalization

The microchips used in this work incorporated a 30 × 4 × 0.2 mm^3^ (length × width × height) microchannel featuring an array of ∼14 400 micropillars (diameter 50 μm, interpillar distance 100 μm from center to center) in a hexagonal lattice (Figure 1). The total surface area of the micropillar array was 672 mm^2^, the total internal volume ∼17 μL (excluding the microchannels connecting to inlet/outlet), and the specific surface area (surface-to-volume ratio) ∼39 mm^–1^. The microchips were made of off-stoichiometric thiol–enes (OSTE) polymer composition as previously described by Tähkä *et al.*(15) and functionalized with biotin prior to use. Briefly, the OSTE prepolymer was prepared by mixing a tetrafunctional thiol (pentaerythritol tetrakis(3-mercaptopropionate), Thiocure PETMP, Bruno Bock, Marschacht, Germany) and a trifunctional “ene” (triallyl-1,3,5-triazine-2,4,6(1H,3H,5H)-trione, 98%, Sigma-Aldrich, St. Louis, MO) monomers at a ratio that yielded a 50% molar excess of thiol functional groups (*i.e.*, 12.5% molar excess of the tetrathiol monomer) in the bulk. The monomer mixture was then poured onto a premade polydimethylsiloxane (Sylgard 184, Down Corning Corporation, Midland, MI) mold, incorporating a negative replica of the micropillar array, and kept under vacuum for ∼5 min before curing the monomer mixture under UV for 5 min (Dymax 5000-EC series UV flood exposure lamp, nominal power 225 mW/cm^2^, Dymax Corporation, Torrington, CT). After curing, the OSTE-based micropillar array was sealed by laminating a planar cover layer of the same composition, prepared in the same manner, on top of the micropillar array, and finalized by an additional UV exposure for 2 min (Dymax 5000-EC). The biotinylation of the micropillar array was achieved by filling the microchannel with 0.1 mg/mL biotin-PEG_4_-alkyne (Sigma-Aldrich) in ethylene glycol, with 1% (m/v) Irgacure TPO-L (BASF, Ludwigshafen, Germany) as the photoinitiator, after which the cross-linking reactions between biotin-PEG_4_-alkyne and the surface thiols were initiated by UV (LED, λ = 365 nm, nominal intensity 14 mW/cm^2^). After UV exposure (1 min), the microchannel was rinsed sequentially with methanol (Sigma-Aldrich) and Milli-Q water (3–5 mL each) and dried before use. The structural fidelity of the micropillar arrays was confirmed by scanning electron microscopy (Quanta 250 FEG, FEI, Hillsboro, OR) using a platinum coating (∼10 nm coating thickness), as exemplified in Supplementary Figure 1.

Before loading the biotinylated pan-HLA antibody (1.6 mg/mL in PBS, BioLegend), the biotin-functionalized micropillars were precoated by filling the micropillar array with streptavidin (0.1 mg/mL in PBS, Sigma-Aldrich), incubating for 15 min, and rinsing with 200 μL of PBS three times. All capillaries (PTFE) and capillary couplings (Nanoport, PEEK) were of inert and biocompatible materials to reduce the risk of cross-contamination and loss of sample.

Whenever fluorescent labeled streptavidin or antibodies were used, the quantitation of the fluorescence signal arising from on-chip immobilized biomolecules was performed through the top layer of the chip using a Zeiss Axioscope A1 upright epifluorescence microscope (Carl Zeiss Oy, Espoo, Finland) equipped with a HAL100W broadband lamp (Carl Zeiss) and a Hamamatsu R5929 photomultiplier tube coupled with a Cairn Integra signal amplifier module (Cairn Research, Faversham, U.K.). The on-chip fluorescence signals (excitation 488 ± 5 nm, emission 500–700 nm) were quantitated and averaged from a total of three locations along the micropillar array. Typically, 3–4 technical replicates (chips) were used in 30% acetonitrile containing 0.1% trifluoroacetic acid, and the effluent was evaporated to dryness by vacuum centrifugation.

### Cell Line and Reagents

The EBV-transformed human lymphoblastoid B-cell line JY (ECACC HLA-type collection, Sigma-Aldrich) was cultured in RPMI 1640 (GIBCO, Invitrogen, Carlsbad, CA) supplemented with 1% GlutaMAX (GIBCO, Invitrogen, Carlsbad, CA) and 10% heat inactivated fetal bovine serum (HI-FBS, GIBCO, Invitrogen, Carlsbad, CA).

Streptavidin (*Streptomyces avidinii*, affinity purified, lyophilized from 10 mm potassium phosphate, ≥13 U/mg protein) was purchased from Sigma-Aldrich (Saint Louis, MO). Biotin-conjugated antihuman HLA-A, B, C clone w6/32 was purchased from Biolegend (San Diego, CA) for analysis. The following peptides were purchased from Ontores Biotechnologies Co., Ltd. and were used throughout the study: KVLEYVIKV (gene name MAGE A1), ILDKKVEKV (gene name HSP90), QLVDIIEKV (gene name PSME3), and LLIENVASL (gene name GPX1). Additionally, the following peptides were purchased from Chempeptide (Shangai, China): VIMDALKSSY (gene name NNMT), FLAEGGGVR (gene name FGA), and EVAQPGPSNR (gene name HSPG2).

### Ovarian Tumor Biopsy and Ethical Consideration

The ovarian tumor biopsy was collected from a patient with ovarian metastatic tumor (high grade serous), who signed an informed consent, under the studies approved by the Research Ethics Committee of the Northern Savo Hospital District with the approval number 350/2020. The samples were chopped in small pieces and treated with a digestion buffer containing collagenase type D (Roche) 1 mg/mL, Hyaluronidase (Sigma-Aldrich) 100 μg/mL and DNase I (Roche) 1 mg/mL for 1 h at 37 °C. The cell suspension was sequentially passed through a 500 and 300 μm cell strainer (pluriSelect) to obtain single cells.

### Renal Cell Carcinoma and Bladder Tumor Samples and Ethical Considerations

Patient tissue samples for organoid cultures were obtained from the DEDUCER study (Development of Diagnostics and Treatment of Urological Cancers) at Helsinki University Central Hospital with approval number HUS/71/2017, 26.04.2017, ethical committee approval number 15.03.2017 Dnro 154/13/03/02/2016, and patient consent. The kidney sample was obtained from a nephrectomy of an adult male with a clear cell renal cell carcinoma (ccRCC, pTNM stage pT3a G2). The benign kidney tissue sample was used for the experiments. The carcinoma urothelial (bladder cancer, high grade, gradus III, 1 cm × 1 cm) was obtained from adult female, and the cancer tissue sample was used for the organoid culture.

### Clear Cell Renal Carcinoma and Bladder Tumor Organoid Culture

Cells were isolated from the original tissue instantly after surgery by dissociating the tissue into small pieces and treating it with collagenase (40 units/mL) for 2–4 h. Benign and cancer cells of the kidney of a clear cell renal cell carcinoma patient cells were grown as organoids in F-medium [3:1 (v/v) of F-12 nutrient mixture (Ham)–DMEM (Invitrogen), 5% FBS, 8.4 ng/mL cholera toxin (Sigma), 0.4 μg/mL hydrocortisone (Sigma), 10 ng/mL epidermal growth factor (Corning), 24 μg/mL adenine (Sigma), 5 μg/mL insulin (Sigma), 10 μM ROCK inhibitor (Y-27632, Enzo Life Sciences, Lausen, Switzerland), and 1% penicillin-streptomycin with 10% Matrigel (Corning). The bladder tumor-derived organoids were grown in hepatocyte calcium medium (Corning)^49^ supplemented with 5% CSFBS (Thermo Fisher Scientific), 10 μM Y-27632 RHO inhibitor (Sigma), 10 ng/mL epidermal growth factor (Corning), 1% GlutaMAX (Gibco), 1% penicillin-streptomycin, and 10% Matrigel. A total of 6 × 10^6^ cells were collected by centrifugation, washed in PBS to remove Matrigel, and snap frozen before analyses.

### HLA Typing

The clinical HLA typing of tumor samples (ccRCC and bladder) was performed by the European Federation for Immunogenetics (EFI)-accredited HLA laboratory of the Finnish Red Cross Blood Service. Allele determination of three classical HLA-I genes, HLA-A, HLA-B, and HLA-C, was performed by the targeted PCR based next generation sequence (NGS) technique according to the protocol provided by the manufacturer (NGSgo Workflow, GenDx, Utrecht, The Netherlands).

The allele assignment at the four-field resolution level was implemented by NGSengine version 2.11.0.11444 (GenDx, Utrecht, The Netherlands) using IPD IMGT/HLA database, release 3.33.0; https://www.ebi.ac.uk/ipd/imgt/hla/.

### Flow Cytometry Analysis

The following antibodies were used to analyze the cell surface expression of HLA-A2 and HLA-A, HLA-B, and HLA-C: PE-conjugated antihuman HLA-A2 (clone BB7.2, BioLegend 343306 San Diego, CA), PE-conjugated antihuman HLA-A, HAL-B, and HLA-C (clone W6/32, BioLegend 311406, San Diego, CA), and Human TruStain FcX block (BioLegend B247182, San Diego, CA).

The data were acquired using a BDLSR Fortessa flow cytometer. Flow cytometric analysis of renal cell carcinoma and bladder tumor-derived organoids was performed using a BD Accuri 6 plus (BD Biosciences) and analyzed with FlowJo software (Tree Star, Ashland, OR).

### Immobilized Biotinylated pan-HLA Antibody Titer Assay

The amount of immobilized biotinylated pan-HLA antibody has been titered comparing the amount of the antibody in the feed solution versus the output solution. In detail, 12.5 μg of anti-panHLA (Biolegend, catalog no. 311434, clone W6/32, biotin conjugated) in 25 μL was added into the microchip at each cycle and incubated for 15 minutes at room temperature. After the incubation time, the microchip was washed three times with 200 μL of PBS and the elute collected. The antibody in the output solution was then quantified by ELISA. Briefly, maxisorb ELISA Nunc plates were coated with the output solution overnight at +4 °C. After washing, 4% BSA (BioTop Oy) in PBS was added and incubated for 2 h at 37 °C, followed by washing steps in 0.05% Tween20 (Sigma-Aldrich). Streptavidin/HRP (Pierce) was added for 30 min, followed by additional washing steps. Finally, TMB (Pierce) solution was applied for 20 min, and sulfuric acid (Sigma-Aldrich) 0.16 M was used to stop the reaction and the plate read at 450 nm. The amount of biotinylated pan-HLA antibody was quantified by extrapolating the signal into a linear range (signal vs concentration) of a standard curve.

### Purification and Concentration of HLA Class I Peptides

HLA class I peptides were immunoaffinity purified from the JY human cell line using biotin-conjugated antihuman HLA-A, HLA-B, and HLA-C antibodies (clone W6/32, BioLegend 311434 San Diego, CA). For sample preparation, the snap-frozen cell pellet was pipetted up and down 20 times in the lysis buffer. The lysis buffer contained 150 mM NaCl, 50 mM TRIS-HCl, pH 7.4, protease inhibitors (A32955 Thermo Scientific Pierce, Waltham, MA), and 1% Igepal (I8896 Sigma-Aldrich, St. Louis, MO). The lysates were first cleared by slow centrifugation for 10 min at 500*g*, and then the supernatant was centrifuged for 30 min at 25 000*g*. Next, HLA-I complexes were immunoaffinity purified from the cleared lysate with antihuman HLA-A, HLA-B, and HLA-C biotin–streptavidin bound to the micropillars of the biotinylated thiol–ene CHIP. The CHIPs were first washed three times with PBS, and then the HLA molecules were eluted at room temperature by adding acetic acid 7% (A113 Fisher Scientific, Leicestershire U.K.) in 50% MeOH (10402824 Fisher Scientific, Leicestershire, U.K.).

Eluted HLA peptides and the subunits of the HLA complexes were desalted using SepPac-C18 cartridges (Waters) according to the protocol previously described by Bassani *et al.*(50) Briefly, the cartridge was prewashed with 80% acetonitrile in 0.1% trifluoroacetic acid (TFA) and then with 0.1% TFA. The peptides were purified from the HLA-I complex by elution with 30% acetonitrile in 0.1% TFA. Finally, the samples were dried using vacuum centrifugation (Eppendorf).

### LC–MS/MS Analysis of HLA Class I Peptides

Each dry sample was dissolved in 10 μL of LC–MS solvent A (0.1% formic acid). The nanoElute LC system (Bruker, Bremen, Germany) injected and loaded the 10 μL of sample directly onto the analytical column (Aurora C18, 25 cm long, 75 μm i.d., 1.6 μm bead size, Ionopticks, Melbourne, Australia) constantly kept at 50 °C by a heating oven (PRSO-V2 oven, Sonation, Biberach, Germany). After washing and loading sample at a constant pressure of 800 bar, the LC system started a 30 min gradient from 0 to 32% solvent B (acetonitrile, 0.1% formic acid), followed by an increase to 95% B in 5 min, and finally a wash of 10 min at 95% B, all at a flow rate of 300 nL/min. Online LC–MS was performed using a Tims TOF Pro mass spectrometer (Bruker, Bremen, Germany) with the CaptiveSpray source, capillary voltage 1500 V, dry gas flow of 3 L/min, and dry gas temperature at 180 °C. MS data reduction was enabled. The mass spectra peak detection maximum intensity was set to 10. The mobilogram peak detection intensity threshold was set to 5000. The mass range was 300–1100 *m*/*z*, and the mobility range was 0.6–1.30 V s/cm^2^. MS/MS was used with 3 PASEF (parallel accumulation–serial fragmentation) scans (300 ms each) per cycle with a target intensity of 20 000 and an intensity threshold of 1000, considering charge states 0–5. Active exclusion was used with release after 0.4 min, reconsidering a precursor if the current intensity is >4-fold the previous intensity, a mass width of 0.015 *m*/*z*, and a 1/*k*_0_ width of 0.015 V s/cm^2^. The isolation width was defined as 2.00 *m*/*z* for mass 700 *m*/*z* and 3.00 *m*/*z* for mass 800 *m*/*z*. The collision energy was set as 10.62 eV for 1/*k*_0_ 0.60 V s/cm^2^ and 51.46 eV for 1/*k*_0_ 1.30 V s/cm^2^. Precursor ions were selected using 1 MS repetition and a cycle overlap of 1 with the default intensities/repetitions schedule.

### Proteomics Database Search

All MS/MS spectra were searched by PEAKS Studio X+ (v10.5 build 20191016) using a target–decoy strategy. The database used was the Swissprot Human protein database (including isoforms, 42 373 entries, downloaded from uniprot.org on 2019-11-26).

A precursor mass tolerance of 20 ppm and a product mass tolerance of 0.02 Da for CID-ITMS2 were used. There was no enzyme, the digest mode was unspecific, and oxidation of methionine was used as a variable modification, with a maximum of three oxidations per peptide. A false discovery rate (FDR) cutoff of 1% was employed at the peptide level. The mass spectrometry proteomics data have been deposited to the ProteomeXchange Consortium *via* the PRIDE partner repository with the data set identifier PXD022194. The data set is currently hidden but will be made public upon eventual acceptance of the current manuscript.

### Algorithms Used for Prediction of Peptide Ligands

Affinity to the corresponding HLA alleles was predicted for all eluted peptides identified in the JY cell line using NetMHC4.0. The threshold for binding was set to rank 0.5% to include only the strong binding partners.

### GIBBS Clustering Analysis

Clustering of peptides into groups based on sequence similarities was performed using the GibbsCluster-2.0 tool with the default setting.

### PBMC Stimulation Protocol

PBMCs from healthy donors were either purchased from Immunospot (Bonn, Germany) or isolated from whole blood of healthy donors using a Ficoll (Merck Millipore) density gradient and cultured in RPMI-1640 supplemented with 10% FBS (Gibco), 1% penicillin-streptomycin (Gibco), 1% GlutaMAX (Gibco), 15 mM HEPES, 50 μM β-mercaptoethanol (Gibco), and 1 mM sodium pyruvate (Gibco). The PBMCs were cultured and stimulated according to the following schedule:

- Day 0: thawing of the PBMCs and addition of 10 ng/mL IL-4, 800 IU/mL GM-CSF, 10 ng/mL IL-7, and 5 ng/mL IL-15.
- Day 2: addition of 10 ng/mL LPS, 50 IU/mL IFN-γ, 10 ng/mL IL-4, 800 IU/mL GM-CSF, 60 ng/mL IL-21, and 5 mg/mL peptides.
- Day 5 and 7: addition of 5 ng/mL IL-15, 5 ng/mL IL-7, 60 ng/mL IL-21, and 5 mg/mL peptides.
- Day 9: addition of 5 ng/mL IL-15, 5 ng/mL IL-7, and 5 mg/mL peptides.

PBMCs from patients were stimulated as previously described^30^ with slight modifications. A total of 0.2 × 10^6^ cells were allocated per well and cultured in RPMI-1640 (GIBCO, Invitrogen, Carlsbad, CA) supplemented with 20% FBS (HI GIBCO, Invitrogen, Carlsbad, CA), 1% penicillin-streptomycin (GIBCO, Invitrogen, Carlsbad, CA), and 1% GlutaMAX (GIBCO, Invitrogen, Carlsbad, CA) and in the presence of IL-4 (Biotechne) and GM-CSF (Biotechne) for 24 h. In total, 1 μM peptide (Chempeptide), 0.5 ng/mL IL-7 (Biotechne), and 20 mg/mL Poly-I:C (Invitrogen) were added after 24 h. The stimulation lasted 9 days.

### CD8^+^ T Cell Isolation

CD8^+^ T cells were isolated by MACS depletion (Miltenyi Biotec, Bergisch Gladbach, Germany) from PBMCs stimulated according to the aforementioned protocol.

### Real-Time Impedance-Based Cytotoxicity Assay

The cytotoxicity assay was performed using the xCELLigence real-time cell analysis system (ACEA Biosciences Inc.). Briefly, 80 000 JY cells or 75 000 ccRCC cells were seeded in a total volume of 50 μL per well (in an antiCD19 precoated 8-well plate in case of the JY) and cultured for 24 h at 37 °C in 5% CO_2_. After 24 h, the effector cells (purified CD8 T cells) were added at a target (E/T) ratio of 1:1. The effector and target cells were cocultured for 36 h, and the CI of the target cells was measured every 1 h. The normalized cell index (NCI) was used for the analysis, and the following formula was applied:
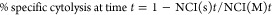
where NCI is the normalized cell index, s is the sample, and M is the mock effector control.

### Bioinformatic Analysis

The functional annotation and visualization was performed by using the clusterProfiler^51^ Bioconductor package (v. 3.12.0) in the RStudio server environment (v. 3.6.0). ClusterProfiler implements a hypergeometric test to evaluate the statistical enrichment of the input gene list over the desired functional classes. Nominal *p*-values were adjusted by applying the Benjamini–Hochberg method,^52^ and the threshold was set to padj = 0.01. The mapping between different human gene identifiers was performed through the use of the org.Hs.eg.db Bioconductor library.^53^ The analysis of the Molecular Signatures Database (MSigDB) (url https://www.gsea-msigdb.org/gsea/msigdb) was performed by using the msigdbr CRAN package, while the visualization of the results was obtained by employing the ComplexHeatmap Bioconductor package.^54^

### Statistical Analyses

Statistical analysis was performed using GraphPad Prism 8.0 software (GraphPad Software Inc.). Details about the statistical tests for each experiment can be found in the corresponding figure legends.

## Supporting Information Available

Scanning electron micrograph of a thiol–ene micropillar array before sealing and functionalization; characterization of microchip functionalization; flow cytometry analysis of the JY cell line; gene ontology enrichment analysis of HLA-I ligands; Molecular Signatures Database analysis of HLA-I ligands; number of unique peptides isolated for PeptiCHIP and the standard method; gene ontology (GO) enrichment analysis of the source proteins found in the ovarian tumor biopsies; flow cytometry analysis of pan-HLA in RCC and bladder tumor PDOs; and unsupervised Gibbs clustering analysis defining the consensus binding motif in RCC and bladder tumor PDOs (PDF)

## Notes

A preprint of this manuscript was made available earlier under the previous title of “PeptiCHIP: A Novel Microfluidic-Based Chip Platform for Tumour Antigen Landscape Identification”, Sara Feola, Markus Haapala, Karita Peltonen, Cristian Capasso, Beatriz Martins, Gabriella Antignani, Antonio Federico, Vilja Pietiäinen, Jacopo Chiaro, Michaela Feodoroff, Antti Rannikko, Manlio Fusciello, Satu Koskela, Jukka Partanen, Firas Hamdan, Sari Tähkä, Erkko Ylösmäki1, Dario Greco, Mikaela Grönholm, Tuija Kekarainen *et al.* March 3, 2021, PREPRINT (Version 1) available at Research Square [10.21203/rs.3.rs-156531/v1], date of access March 4, 2021.
