# Supplementary material for: PeptiCHIP: A Microfluidic Platform for Tumor Antigen Landscape Identification
Source: ACS Nano. 2021 Oct 4;15(10):15992–6010. doi: 10.1021/acsnano.1c04371 (PMC8552492; doi:10.1021/acsnano.1c04371)
Supplement: Supplementary file 1 — nn1c04371_si_010.pdf [file nn1c04371_si_010.pdf]

PeptiCHIP: A Microfluidic Platform for Tumor Antigen Landscape Identification

Sara Feola[1,2,3,4,], Markus Haapala[5], Karita Peltonen[1,2,3,4], Cristian Capasso[1,2,3,4], Beatriz Martins[1,2,3,4], Gabriella Antignani[1,2,3,4], Antonio Federico[6], Vilja Pietiäinen [2,4,7], Jacopo Chiaro[1,2,3,4], Michaela Feodoroff[, 1,2,3,4,7] ,Salvatore Russo [1,,2,3,4], Antti Rannikko [4,8,9], Manlio Fusciello[1,2,3,4], Satu Koskela[10] , Jukka Partanen[10] , Firas Hamdan[1,2,3,4], Sari M. Tähkä[5], Erkko Ylösmäki[1,2,3,4,], Dario Greco[6], Mikaela Grönholm[1,2,3,4,], Tuija Kekarainen[11], Masoumeh Eshaghi[11], Olga L. Gurvich[11], Seppo Ylä-Herttuala[12], Rui M M Branca[13], Janne Lehtiö[13], Tiina M. Sikanen[5], Vincenzo Cerullo*[1,2,3,4,14].

Affiliations:

1 Drug Research Program (DRP) ImmunoViroTherapy Lab (IVT), Division of Pharmaceutical Biosciences, Faculty of Pharmacy, Viikinkaari 5E, University of Helsinki, 00790 Helsinki, Finland.

2 Helsinki Institute of Life Science (HiLIFE), Fabianinkatu 33, University of Helsinki, 00710 Helsinki, Finland.

3 Translational Immunology Program (TRIMM), Faculty of Medicine Helsinki University, Haartmaninkatu 8, University of Helsinki, 00290 Helsinki, Finland.

4 Digital Precision Cancer Medicine Flagship (iCAN), University of Helsinki, 00014 Helsinki, Finland.

5 Drug Research Program, Division of Pharmaceutical Chemistry and Technology, Faculty of Pharmacy, University of Helsinki, Viikinkaari 5E, 00790, Helsinki, Finland.

6 Faculty of Medicine and Health Technology, Tampere University, Tampere, Arvo Ylpön katu 34, 33520, Finland.

7 Institute for Molecular Medicine Finland, FIMM, Helsinki Institute of Life Science (HiLIFE), University of Helsinki, Biomedicum 2U Tukholmankatu 8, 00290, University of Helsinki, Finland.

8 Department of Urology, Helsinki University and Helsinki University Hospital, Haartmaninkatu 8, 00029, Helsinki, Finland.

9 Research Program in Systems Oncology, Faculty of Medicine, University of Helsinki, Haartmaninkatu 8,00029, Helsinki, Finland

10 Research & Development Finnish Red Cross Blood Service Helsinki, Kivihaantie 7, 00310, Helsinki, Finland.

11 Kuopio Center for Gene and Cell Therapy, Microkatu 1S, 70210 Kuopio, Finland

12 A.I. Virtanen Institute, University of Eastern Finland, Neulaniementie 2, 70211, Kuopio, Finland.

13 Science for Life Laboratory, Department of Oncology-Pathology, Karolinska Institutet, Tomtebodavagen 23B, 171 21, Solna, Sweden.

14 Department of Molecular Medicine and Medical Biotechnology, Naples University “Federico II”, S. Pansini 5, 80131, Naples Italy.

***Corresponding author:**

Vincenzo Cerullo (V.C.): Email: vincenzo.cerullo@helsinki.fi Tel.: 0294159328; 0503185754

The Supporting Information contains the supplementary figures and the supplementary figures legends. Scanning electron micrograph of a thiol-ene micropillar array before sealing and functionalization. Characterization of microchip functionalization. Flow cytometry analysis of JY cell line. Gene Ontology enrichment analysis of HLA-I ligands. Molecular Signatures Database analysis of HLA-I ligands. Number of unique peptides isolated for PeptiCHIP and standard method. Gene Ontology (GO) enrichment analysis of the source proteins found in the ovarian tumor biopsies. Flow cytometry analysis of pan-HLA in RCC and Bladder tumor PDOs. Unsupervised Gibbs clustering analysis defined the consensus binding motif in RCC and Bladder tumor PDOs.

Supporting information:

Supplementary figure 1

Supplementary figure 2

Supplementary figure 3

Supplementary figure 4

Supplementary figure 5

Supplementary figure 6

Supplementary figure 7

Supplementary figure 8

Supplementary figure 9

Table 1

Table 2

Supplementary Figures and Supplementary Figures legends

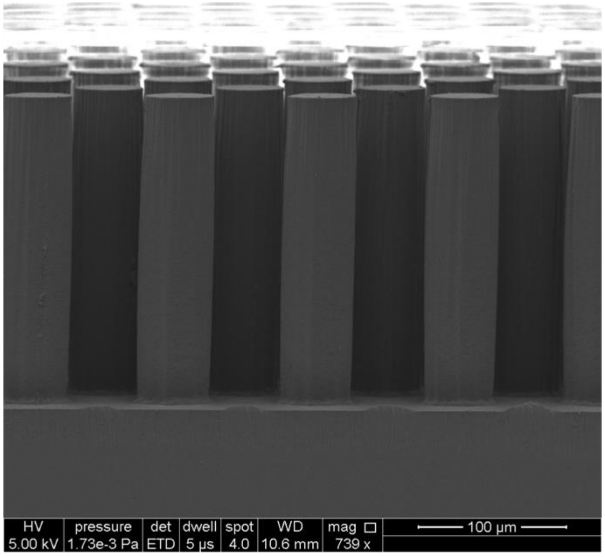

Supplementary Figure 1 Scanning electron micrograph of a thiol-ene micropillar array before sealing and functionalization.

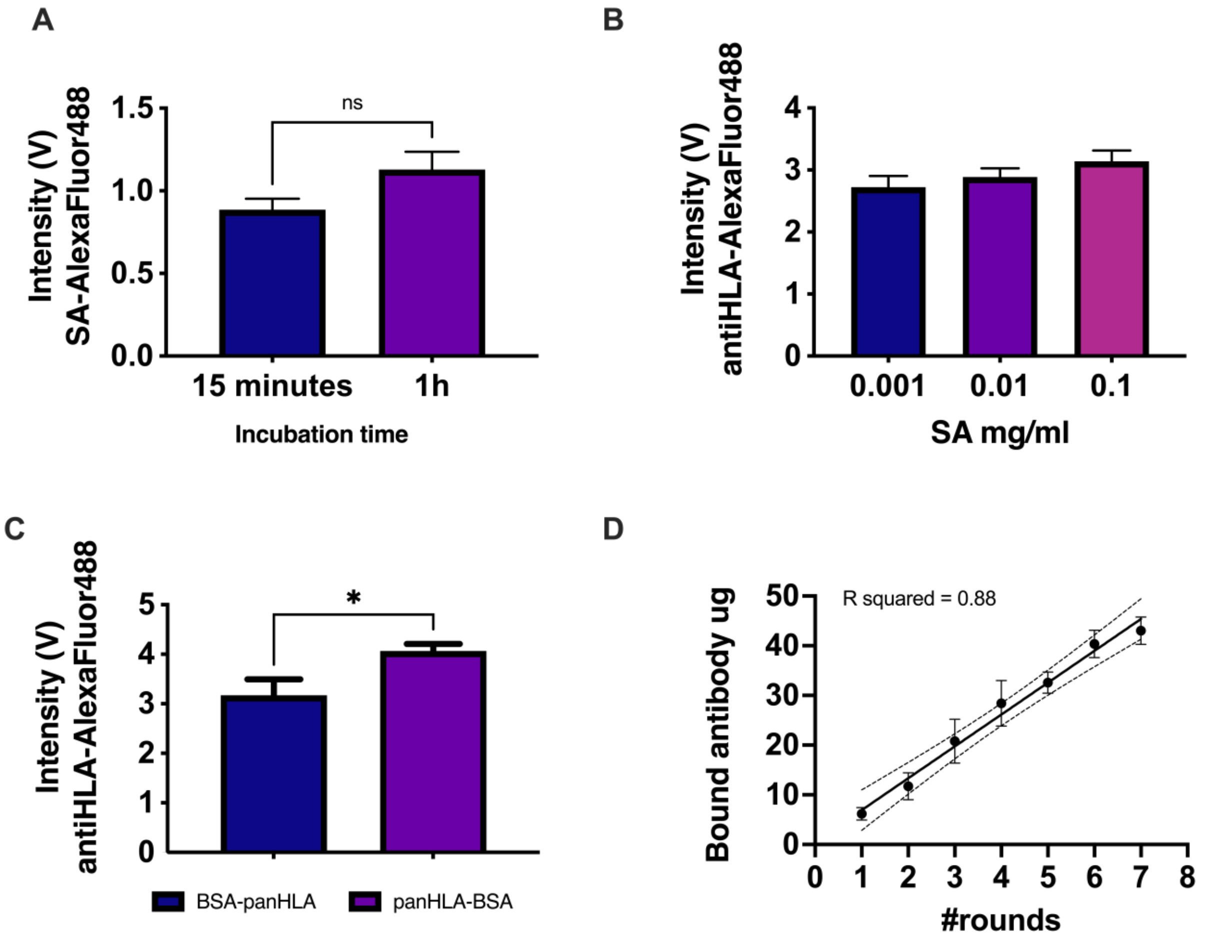

Supplementary Figure 2 Characterization of the selectivity of the microchip functionalization with biotinylated pan-HLA antibody.

A) Binding efficacy of AlexaFluor 488-streptavidin on thiol-ene micropillars precoated with biotin-PEG$_4$-alkyne at two different streptavidin incubation times (15 minutes and 1h). **B)** The effect of streptavidin (nonfluorescent) concentration on the amount of immobilized biotinylated pan-HLA antibody quantitated through AlexaFluor 488-labeled secondary antibody. **C)** The effect of BSA

incubation on the amount of immobilized biotinylated pan-HLA antibody quantitated through AlexaFluor 488-labeled secondary antibody. The efficiency of BSA in blocking nonspecific binding sites was assessed by preconditioning the micropillar array with BSA either before (BSA-panHLA) or after (panHLA-BSA) immobilization of the biotinylated pan-HLA antibody. **D)** The total amount of biotinylated pan-HLA antibody bound onto a single chip as a function of loading cycles. For each cycle, a fresh batch of the same (constant) pan-HLA antibody concentration was used. Significance was assessed by two-tailed unpaired Student's t-test, * $p<0.05$. All the technical characterization was performed at least two time in two separate experiment and in each experiment at least triplicates for each group was performed.

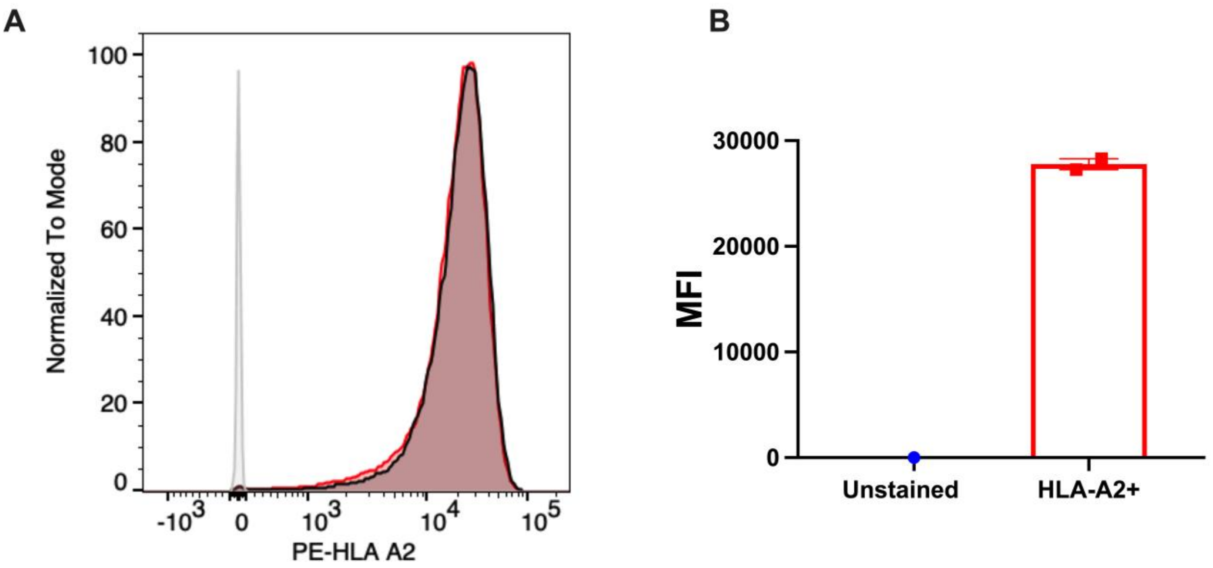

Supplementary Figure 3 Flow cytometry analysis of JY cell line. The data are plotted as bar graphs. **A)** The frequency and **B)** the geometric mean fluorescence intensity (gMFI) for PE-HLA-A2 is showed.

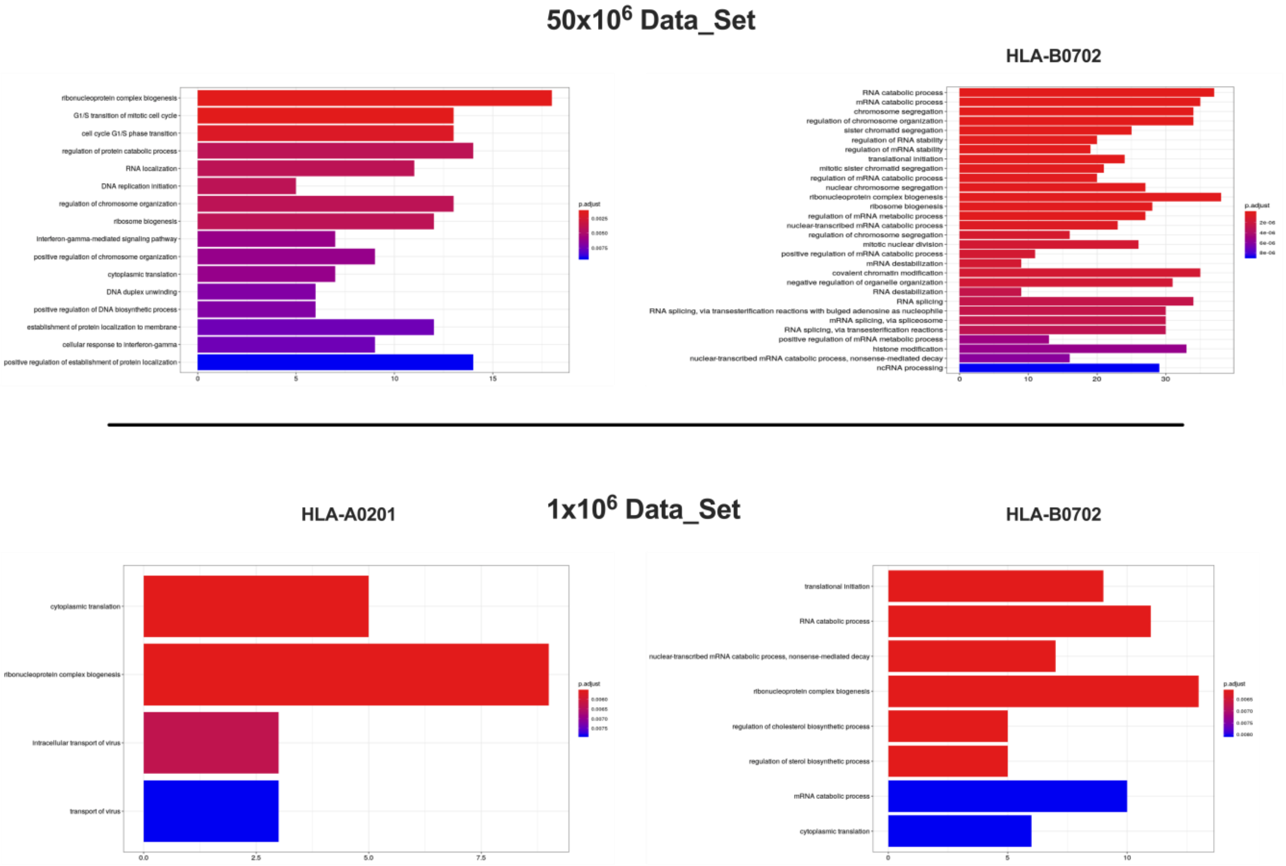

Supplementary Figure 4 Gene Ontology (GO) enrichment analysis of the HLA-ligands source proteins. The most overrepresented biological processes for 50x10^6 cells (upper panel) and for 1x10^6 cells (lower panel) are separately shown for HLA-A*02:01 and HLA-B*07:02 alleles (hypergeometric test padj<0.01).

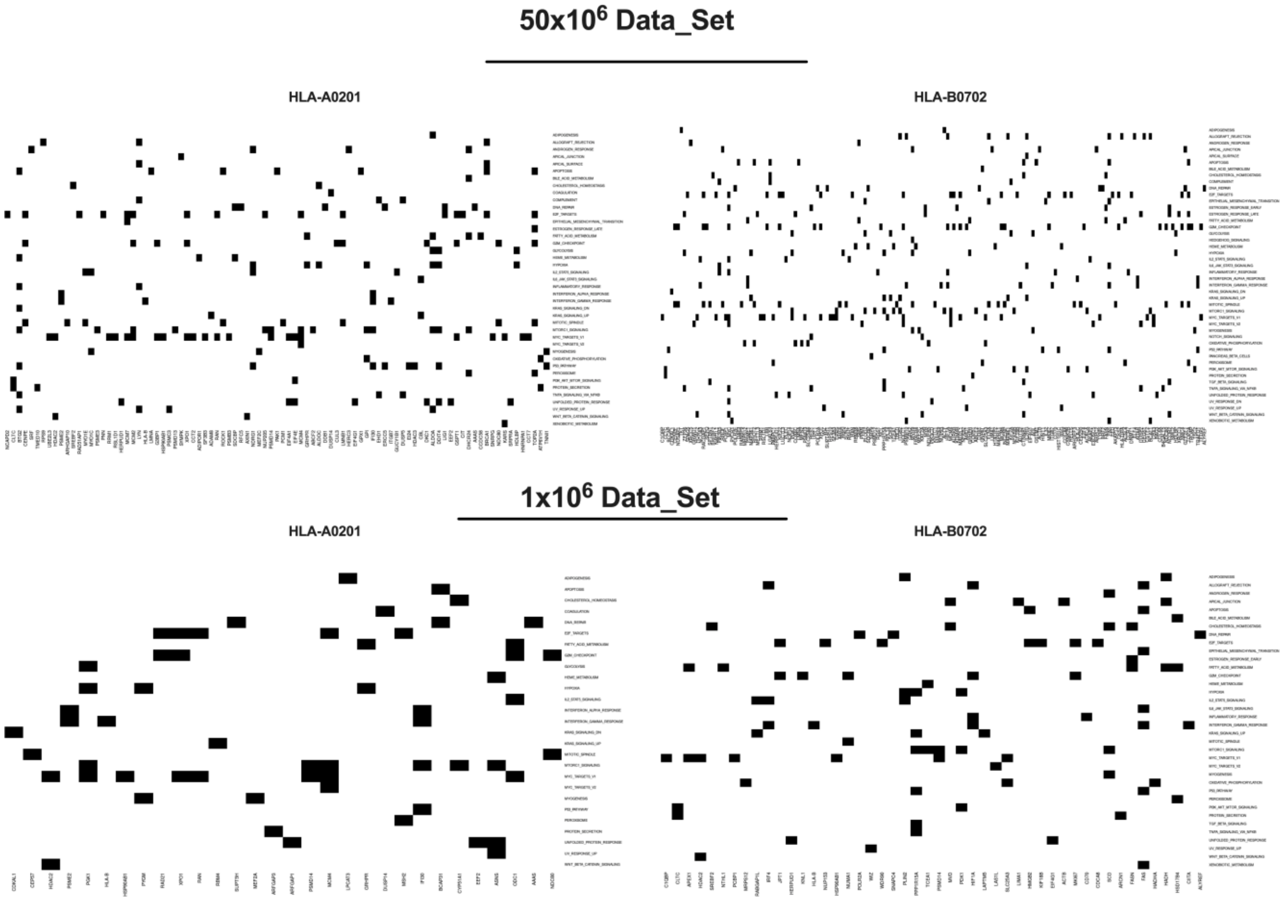

Supplementary Figure 5 Molecular Signatures Database results are showed. The source proteins analysis was performed against the hallmark data set and the results are depicted separately for HLA-A*02:01 and HLA-B*07:02 alleles in both data sets derived from $50x10^6$ cells (upper panel) and for $1x10^6$ cells (lower panel).

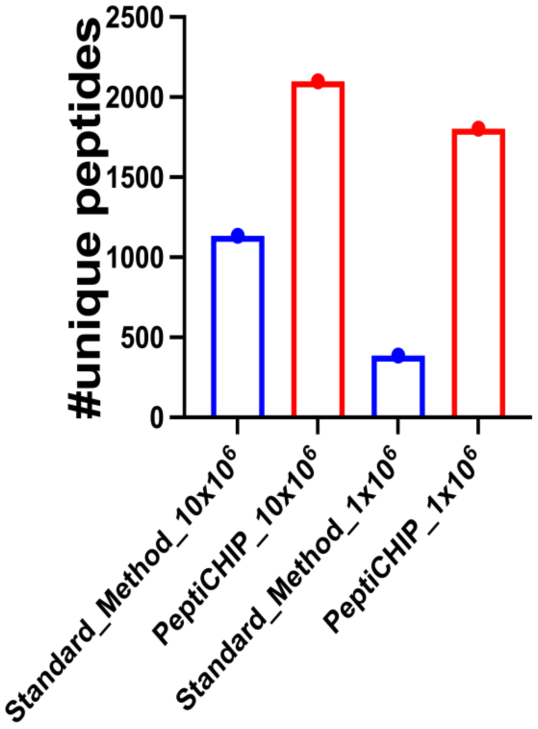

Supplementary Figure 6 The number of unique peptides isolated for each method and for each cell amount is depicted as bar plot.

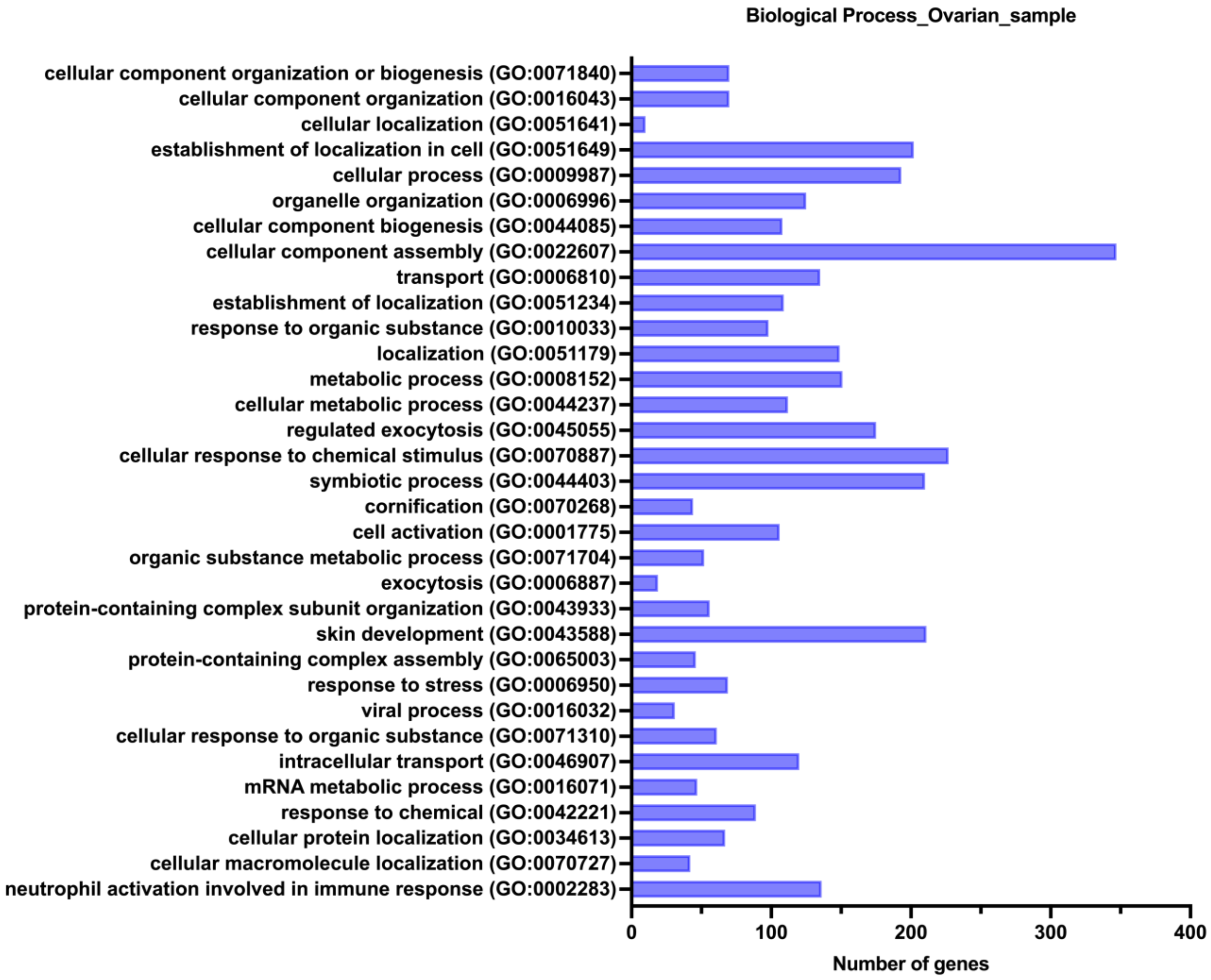

Supplementary Figure 7 Gene Ontology (GO) enrichment analysis of the source proteins found in the ovarian tumor biopsies. Here the biological process overrepresented are shown. The Fisher test with Bonferroni correction and p<0.01 were applied.

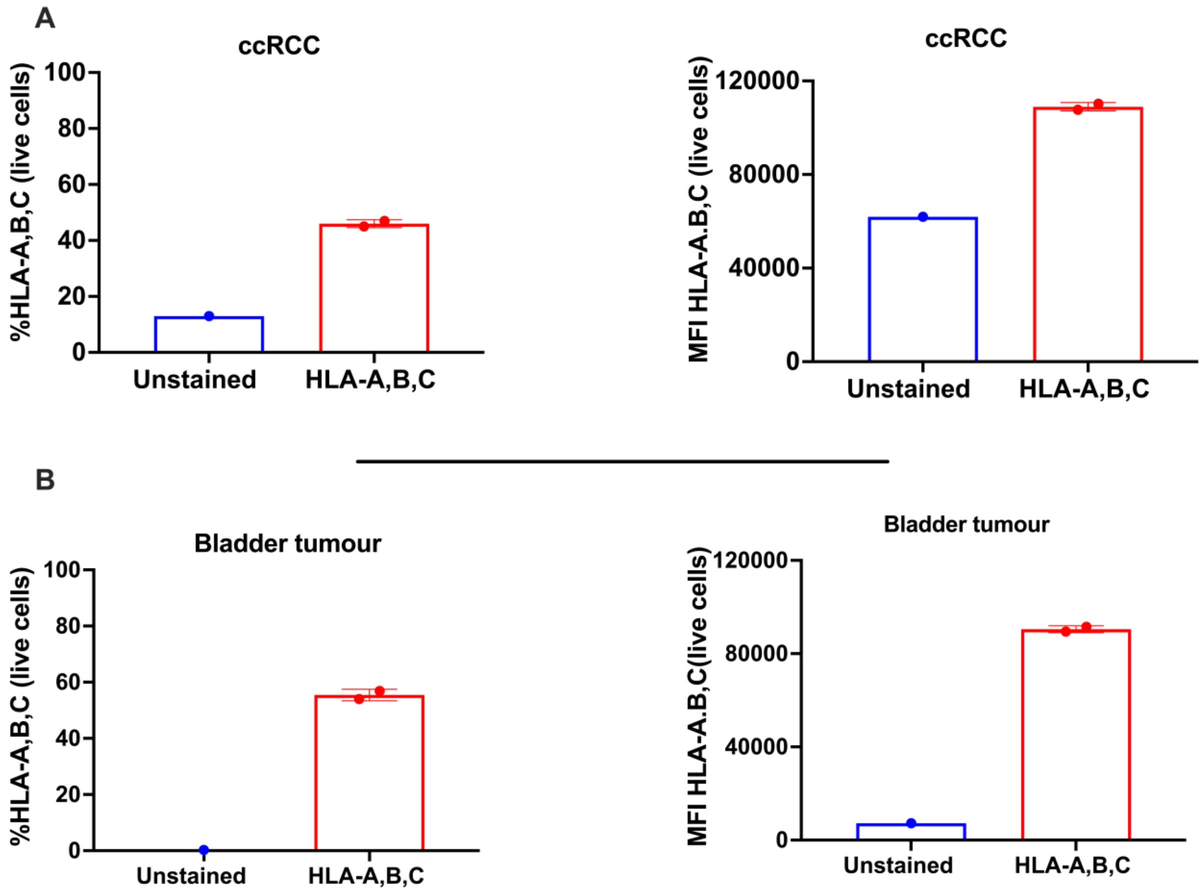

Supplementary Figure 8 Flow cytometry analysis of pan-HLA in RCC and Bladder tumor PDOs. The frequency and the geometric mean fluorescent intensity (gMFI) are shown for both **A)** ccRCC and **B)** bladder tumor PDOs. The data are plotted as bar graphs ± SD.

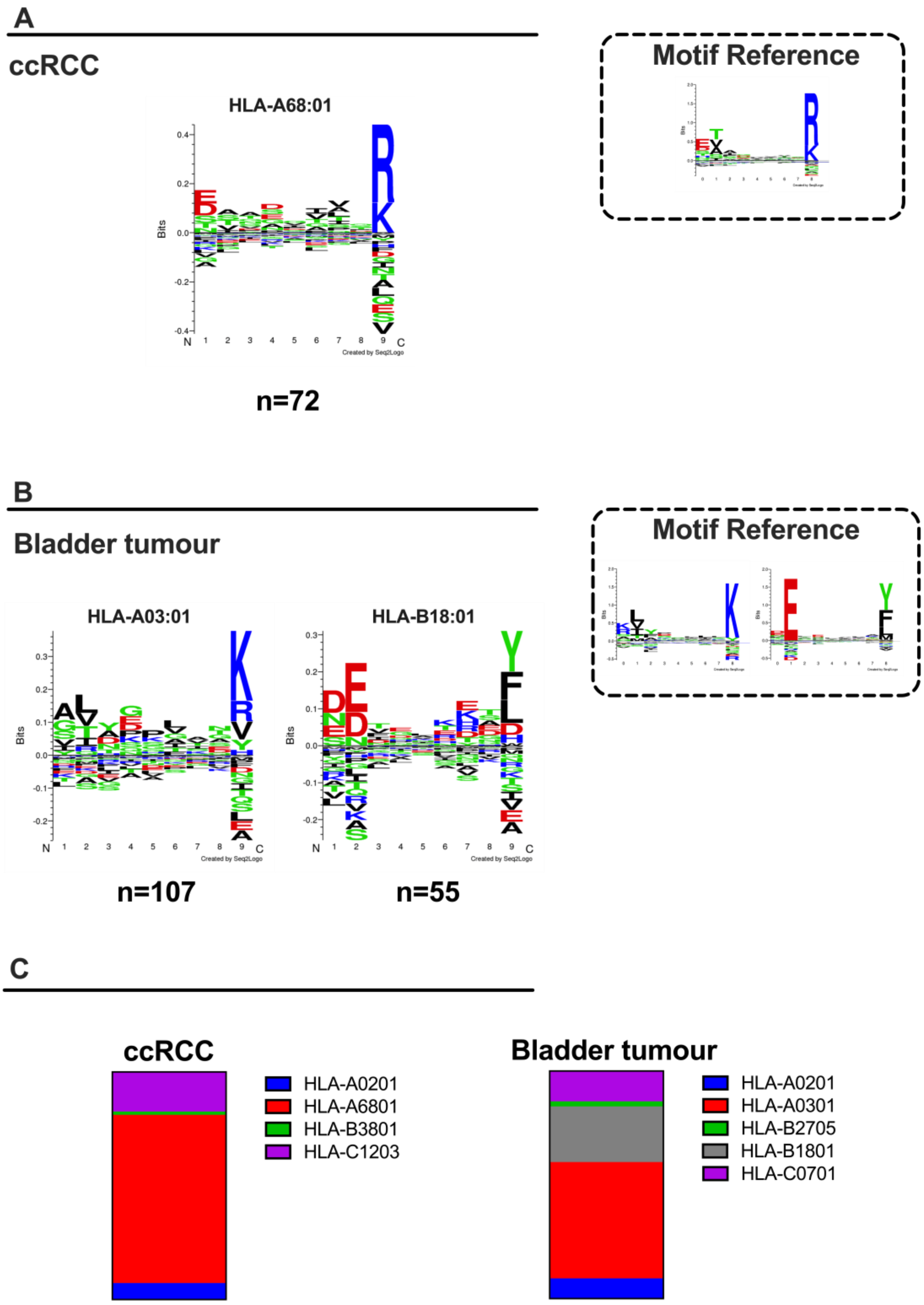
A
ccRCC
HLA-A68:01
Bits
n=72
Motif Reference
B
Bladder tumour
HLA-A03:01
HLA-B18:01
n=107
n=55
Motif Reference
C
ccRCC
HLA-A0201
HLA-A6801
HLA-B3801
HLA-C1203
Bladder tumour
HLA-A0201
HLA-A0301
HLA-B2705
HLA-B1801
HLA-C0701

Supplementary Figure 9 Unsupervised Gibbs clustering analysis defined the consensus binding motif in **A)** RCC and **B)** Bladder tumor PDOs. **C)** NetMHC 4.0 Server was used to predict the binders among the eluted 9mers for an applied rank 2%. The data are showed as a stacked bar chart and each colour describe a specific HLA allele.
